# Anticipating incomplete patient-reported outcomes in schizophrenia: a machine learning approach to predict the occurrence of missing data

**DOI:** 10.1186/s12955-026-02511-1

**Published:** 2026-03-14

**Authors:** Guillaume Barbalat, Julien Plasse, Isabelle Chéreau-Boudet, Benjamin Gouache, Emilie Legros-Lafarge, Nathalie Guillard-Bouhet, Nicolas Franck

**Affiliations:** 1 Centre Ressource de Réhabilitation Psychosociale et de Remédiation Cognitive (CRR), Hôpital Le Vinatier, Centre National de la Recherche Scientifique (CNRS UMR 5229, et Université Lyon 1, Lyon, France; 2 Unité de Recherche Clinique Pierre Deniker, Centre Hospitalier Henri Laborit, CHU et faculté de médecine de Poitiers, Poitiers, France; 3 Centre Référent Conjoint de Réhabilitation (CRCR), Centre Hospitalier Universitaire de Clermont-Ferrand, Clermont-Ferrand, France; 4 Centre Référent de Réhabilitation Psychosociale et de Remédiation Cognitive (C3R), Centre Hospitalier Alpes Isère, Grenoble, France; 5 Centre Référent de Réhabilitation Psychosociale de Limoges (C2RL), Limoges, France; 6 Centre de REhabilitation d’Activités Thérapeutiques Intersectoriel de la Vienne (CREATIV), Centre Hospitalier Laborit, Poitiers, France

**Keywords:** Missing data, Patient-reported outcomes (PROs), Schizophrenia, Psychosocial rehabilitation, Machine learning, Ensemble algorithm, SHAP values, REHABase

## Abstract

**Background:**

Missing data in patient-reported outcome (PRO) databases is a pervasive challenge, particularly in psychiatry and psychosocial rehabilitation. Incomplete data may reduce the generalizability of findings and introduce selection bias. It may also signal loss of access to care, potentially hindering recovery and rehabilitation efforts. Proactively anticipating missingness can help mitigate this issue by identifying individuals at risk of missing values, enabling services to take timely and targeted actions in response. However, the predictability of incomplete PRO data remains underexplored.

**Methods:**

We used data from the French multicentric psychosocial rehabilitation database REHABase, focusing our analysis on patients with schizophrenia. We developed an ensemble machine learning model to predict missing data occurrence across six PROs, incorporating treatment center affiliation, sociodemographic features and clinical predictors. To ensure interpretability, we applied the concept of Shapley values to quantify individual predictor contributions to missing data patterns.

**Results:**

Our sample comprised *N* = 2,363 participants. Averaged areas under the receiving operating curve (AUC) measured on the holdout testing observations ranged from 0.73 to 0.78 across the six PRO scales, demonstrating good predictive performance of our ensemble model. Treatment center affiliation emerged as a critical predictor of missing data. The ten most influential patient-level predictors were: being a disabled worker beneficiary; educational attainment; housing status; duration of illness; antipsychotic medication; origin of the referrer; number of suicide attempts; addictions comorbidity; having a forensic history; and sex. We also identified directional contributions distinguishing positive (increased likelihood of missing values) and negative effects (decreased likelihood).

**Conclusions:**

To our knowledge, this work represents the first predictive analytics framework for the occurrence of missing PRO data in psychosocial rehabilitation. Our ensemble algorithm holds dual potential: improving data collection strategies and informing targeted interventions to enhance patient engagement and retention. By proactively identifying at-risk individuals and refining study designs, our model could also indirectly support better functional recovery outcomes for schizophrenia patients.

**Supplementary Information:**

The online version contains supplementary material available at 10.1186/s12955-026-02511-1.

## Background

Patient-reported outcomes (PROs) are direct reports from patients about their health status, quality of life, or functional ability. They provide essential information for both patients and clinicians, aiding in healthcare decision-making and playing a growing role in clinical research, service evaluation, and performance measurement.

While PROs offer valuable insights, their reliability and accuracy can be significantly compromised by missing data, often resulting from patient withdrawal or loss to follow-up. This poses at least two significant challenges. First, from a research perspective, individuals with incomplete information often differ systematically from those with complete data, potentially reducing the generalizability of future analyses and introducing selection bias [1–4]. Second, non-response may indicate loss of access to care, potentially impeding recovery and rehabilitation efforts.

To our knowledge, much of the research investigating risk factors for missing PRO data stems from cancer studies [5–9] or elective surgery [10–12]. A systematic review identified factors associated with missing PRO data [6], categorizing them as instrument-related (e.g., clarity of language), patient-related (e.g., cognitive issues or personality traits), center-related (e.g., activity level and resources), staff-related (e.g., commitment, administration errors), or study-related (e.g., data collection frequency). These findings were generally corroborated by a cohort study [13] and qualitative interviews with research assistants [5]. The latter specifically incriminated employment, difficulty reading, lack of education, cognitive dysfunctions, being on medication, and physical issues.

Palmer et al. found additional associations between missing PRO data and factors such as age, location, well-being, and more advanced disease stages [7]. No associations were observed for employment or insurance status, and there was no evidence linking missing data to race/ethnicity, gender, caregiver status, cognitive status, physical impairment, treatment and its toxicity. In addition, Nielsen et al. suggested that differences between respondents and non-respondents might be related to the value of the missing patient-reported outcome itself [9]. For instance, patients with declining health tend to respond less frequently to PRO questionnaires.

Despite these advancements, determinants of missing data are infrequently reported in the literature. Studies demonstrating the extent of associations in models using center- and patient-specific characteristics are even rarer. Furthermore, no studies have employed state-of-the-art predictive analytics to optimize the prediction of missing data occurrence. Such methods generally utilize flexible algorithms that account for interactions and non-linearities while minimizing overfitting.

Another significant gap remains in the literature, as no studies have specifically investigated risk factors for non-response among psychiatric populations, particularly those with schizophrenia. This population faces unique challenges that contribute to missing data. Firstly, cognitive impairments and social isolation, common in schizophrenia, may significantly hinder participation in data collection. Secondly, patients with schizophrenia often experience stigma and self-stigmatization, coupled with poor quality of life and functional outcomes [14–16], which can further compromise their rehabilitation progress and willingness to engage in assessments. Extrapolating findings from other medical fields, such as cancer or surgery research, to patients with schizophrenia in rehabilitation settings may be problematic due to potential context-specific associations that could significantly affect prediction quality.

Understanding the characteristics of patients with schizophrenia associated with missing data can inform tailored interventions and follow-up strategies to reduce attrition, improve future study designs, and enhance data collection methods. As mentioned above, this understanding can also impact patient care. Better knowledge of risk factors for missing data can improve patient engagement and retention in mental health programs, potentially leading to enhanced outcomes, i.e. better recovery and functioning.

In the current study, we used state-of-the-art machine learning techniques to predict the occurrence of missing values in self-reported outcomes among patients with schizophrenia in rehabilitation settings. In addition, we sought to identify the primary variables predictive of missingness and to determine whether these predictors were consistent across different scales.

## Methods

### Data

#### Description of the database

We used data from the French multicentric psychosocial rehabilitation database REHABase [17]. Our network comprises 28 psychosocial rehabilitation centers. Patients enrolled in these centers are diagnosed with serious mental illness and are referred through public mental health services, private psychiatrists, general practitioners, or self-referral. Referrals are typically accepted following clinical assessment, provided that patients demonstrate minimal clinical stability and clear motivation to participate in psychosocial rehabilitation programs. Acceptance is not based on the severity of functional impairment. Upon admission, patients undergo a standardized sociodemographic, clinical, functional, and cognitive evaluation conducted by a multidisciplinary team (psychiatrists, nurses, neuropsychologists, occupational therapists, and social workers). These data are recorded in an electronic case report form. Patients then receive a personalized rehabilitation care plan, lasting from several months to a year, aimed at improving daily living skills, promoting employment opportunities, enhancing quality of life, and supporting recovery. Monthly group meetings are held to ensure quality control and maintain interrater reliability. Data collected upon referral includes three kinds: background data (age, sex, clinical history, etc.) collected by physicians; rehabilitation outcome data (well-being, quality of life, etc.) collected by nurses; and neuropsychological data (IQ, attention tests, etc.) collected by neuropsychologists. The database obtained the authorizations required under French legislation (French National 429 Advisory Committee for the Treatment of Information in Health Research, 16.060bis; French 430 National Computing and Freedom Committee, DR-2017-268).

For the purpose of this study, we focused our analysis exclusively on background and rehabilitation outcome data from patients diagnosed with schizophrenia. The initial dataset comprised information from twenty-four clinical centers. To ensure statistical robustness, we consolidated centers with fewer than 50 observations into a single group, resulting in a final set of 11 analytical units, comprising ten individual centers and one grouped category.

#### Dependent variables (missing outcome data)

We examined six distinct missing PRO measures: quality of life (using the Schizophrenia Quality of Life questionnaire – SQoL18 [18], 18 items); well-being (Warwick-Edinburgh Mental Well-being Scale – WEMWBS [19], 14 items); self-stigmatization (Internalized Stigma of Mental Illness – ISMI [20], 29 items); self-esteem (Self-Esteem Rating Scale – SERS [21], 20 items); medication adherence (Medication Adherence Rating Scale – MARS [22], 10 items); and insight (Birchwood Insight scale – BIS [23], 8 items). All scales are validated self-report questionnaires commonly used in studies of schizophrenia. For these questionnaires, the percentage of missing values varied from 31.7% (WEMWBS) to 43.5% (MARS).

#### Predictors (background data)

Among the numerous variables available in REHABase, the selection was conducted by clinicians (ICB, BG, ELL, NGB, NF) based on their expertise in psychosocial rehabilitation. The goal was to retain as many variables as possible while satisfying two criteria: [1] data availability (i.e., variables with excessive missing values were excluded); and [2] temporality (i.e., predictors measured at or after the assessment of PROs were not retained). It should also be noted that information such as motivation, personality traits, and race/ethnicity was not available in the database. Predictors of missing rehab outcome data included the following background clinical and socio-demographic factors: centre (11 categories); age (continuous); sex (male vs. female); education (no high school diploma; high school diploma; Bachelor’s degree; Master’s degree); marital status (single; divorced/widowed; in a relationship); being a parent (yes vs. no); housing status (homeless; group home; family home; personal residence); employment (employed (regular); employed (specialized); unemployed); being a disabled worker beneficiary (yes vs. no); being of no fixed abode (currently; in the past; no); duration of illness (less than two years; two to five years; five to 10 years; more than 10 years); number of psychiatric admissions (nil; one; two; three; four; five to 10; more than 10); total time spent in hospital (nil; three months or less; three to six months; six to 12 months; more than 12 months); psychiatric comorbidities (yes vs. no); physical comorbidities (yes vs. no); addiction comorbidities (nil; behavioral only; substance only; both substance and behavioral); history of suicide attempts (no previous attempt; one; two; three; four or more); forensic history (yes vs. no); antipsychotic medication (nil; first-generation antipsychotics; second-generation antipsychotics; both); somatic medication (yes vs. no); and referrer (clinician from the public healthcare system; clinician from the private healthcare system; social worker; self-referral; other).

### Analysis

### Strategy for handling missing data in the predictors set

While we aimed to predict the occurrence of missing data in psychosocial rehabilitation scales, our matrix of predictors itself was not exempt of missing values. The overall percentage of missing values was 4.2%, ranging from 0% (center, age and sex) to 8.5% for duration of illness, 10.0% for number of psychiatric admissions, and 19.5% for total time spent in hospital.

Discarding incomplete data and performing a complete case analysis risked biasing our sample towards those participants without missing information. Therefore, our general strategy aimed to retain as many observations and variables as possible. We applied three predetermined criteria to decide whether to exclude observations or variables from the dataset. First, we excluded observations that consisted entirely of missing values; however, no observations met this criterion. Second, we evaluated variables using the outflux-influx plot, which visualizes the connectivity of variables in a dataset with missing data [24]. Outflux represents how a variable’s observed data relate to missing data in other variables, while influx reflects how a variable’s missing data connect to observed data in others. Variables with low outflux and high influx are typically removed, as they contribute little information while requiring extensive imputation. Based on this assessment, all variables were retained. Third, we used the fraction of missing information (FMI), which quantifies the impact of missing data on parameter estimation in general linear modeling [25]. The FMI ranges from 0 to 1, with higher values indicating a greater impact on estimation precision. Variables with substantially higher FMI values compared to others were excluded. Most variables had an FMI below 0.15, except for two: number of psychiatric admissions and total duration of admission, which had FMI values between 0.58 and 0.59. After excluding these two variables, the FMI values for the remaining variables were generally below 0.10, with two exceptions: duration of illness less than 2 years (FMI = 0.12) and referrer origin: social worker (FMI = 0.19).

We generated m = 30 imputed datasets, acknowledging that no formal guidelines exist for determining the optimal number of imputations in such analyses. To ensure methodological rigor, imputation was performed prior to predictive modeling and conducted separately on the training and hold-out testing sets to prevent data leakage (see below).

#### Predictive modeling

We employed a SuperLearner ensemble model using the *SuperLearner* R package to optimize predictions of the occurrence of missing outcome data (classification task) [26]. Basis learners included general linear modeling with main terms only, general linear modeling with first-order interactions, regularized regression, naive Bayes, random forest, and extreme gradient boosting. We used an “adaptive” hyperparameter tuning strategy [27] aiming to minimize logarithmic loss.

In addition to training each basis learner on the full set of predictors, variables pre-selection was performed using: Chi-square test (*p* < 0.2) for categorical variables and Pearson correlation for age (continuous) (*p* < 0.2); random forest variable importance (to select the top 10 predictors by mean decrease in impurity; implemented with 1000 trees; mtry = 7; nodesize = 1). Each basis learner was used both with and without the variable preselection process, except for the generalized linear model with first-order interactions, which was applied only after variable preselection to avoid excessive computational time. In total, 17 basis learners were included, in addition to the “mean” learner, which predicts outcomes using the average probability of missingness across all observations. For each outcome, data were split into training (70%) and hold-out testing (30%) sets. Base learners and the ensemble model were trained on the training set using 8-fold cross-validation, preserving outcome class distributions across folds. Feature preselection was performed automatically within each fold and imputed dataset. Consequently, the set of features retained may differ across folds and imputations due to data-driven selection, reflecting inherent variability in the modeling process. Model performance was assessed on the hold-out testing set for each of the 30 imputed datasets using two metrics: the area under the receiver operating characteristic curve (AUC) and accuracy.

To enhance the robustness of our approach, we employed a 10-fold cross-validation procedure instead of a single train/test split. To limit computational demands, we generated a single imputed dataset using a random forest algorithm.

#### Variable importance

We computed SHAP (SHapley Additive exPlanations [28]) values for every feature and training observation across all 30 imputed datasets using the *fastshap* R package [29]. For a given observation, SHAP values quantify the magnitude and direction (positive/negative) of each feature’s additive contribution to the model’s predicted likelihood of a missing PRO. Specifically, positive SHAP values indicate a feature increases the likelihood of missing data. Negative SHAP values indicate a feature reduces the probability of missingness. The magnitude reflects the relative importance of each feature’s contribution. By design, the sum of all SHAP values for an observation, combined with the model’s base value (i.e., the average prediction), equals the model’s output for that observation.

To analyze feature importance, we generated two visualizations using the *shapviz* R package: (1) global feature importance via mean absolute SHAP values; (2) directional contributions distinguishing positive (increased likelihood of missing values) and negative effects (decreased likelihood). We present feature importance plots for two PRO scales, the SQoL18 and the MARS, in the main text. Plots for additional scales are included in the Supplementary Materials for comprehensive reference.

## Results

### Sample characteristics

Our final sample comprised *N* = 2,363 participants. The number of participants per analytical unit ranged from 64 to 698, representing 2.7% to 30% of the total sample, respectively. 1,752 participants were male (74%). The median age was 31 years old (Q1, Q3: 24, 40). Regarding education, 47% of participants had no high school diploma, while 6% had completed a Master’s degree. Comorbidities were common, with 55% of participants having an addiction comorbidity and 25% having a physical comorbidity. Most participants (81%) were single, and 74% were referred by a clinician from the public healthcare system. In terms of living arrangements, 44% resided in a personal residence. The majority (89%) were unemployed, and 35% were disabled worker beneficiaries. A significant portion (42%) had been ill for ten years or more. Notably, 4.5% of participants were not taking any antipsychotic medications. A comprehensive description of our raw sample is presented in Table 1, with information on imputed datasets available in Supplementary Table 1.

**Table 1:** Characteristics of the population

Characteristic	*N* = 2,363^*1*^
**Sex**	
Female	611 (26%)
Male	1,752 (74%)
**Age**	31 (24, 40)
**Education**	
No dipl.	1,107 (47%)
HS dipl.	702 (30%)
Bach.	372 (16%)
Mast.	141 (6.0%)
(Missing)	41 (1.7%)
**Marital Status**	
In a rel.	273 (12%)
Div./Wid.	109 (4.6%)
Single	1,910 (81%)
(Missing)	71 (3.0%)
**Children**	
No	2,027 (86%)
Yes	263 (11%)
(Missing)	73 (3.1%)
**Housing**	
Gp H.	220 (9.3%)
Fam. H.	934 (40%)
Pers. H.	1,042 (44%)
Hless	92 (3.9%)
(Missing)	75 (3.2%)
**No Fixed Abode**	
No	2,010 (85%)
Past	184 (7.8%)
Current	74 (3.1%)
(Missing)	95 (4.0%)
**Employment**	
Empl.(reg.)	129 (5.5%)
Empl.(spec.)	48 (2.0%)
Unempl.	2,094 (89%)
(Missing)	92 (3.9%)
**Dis. Work. Benef.**	
No	1,357 (57%)
Yes	824 (35%)
(Missing)	182 (7.7%)
**Antipsychotics**	
Nil	106 (4.5%)
FGA	181 (7.7%)
SGA	1,498 (63%)
Both	545 (23%)
(Missing)	33 (1.4%)
**Dur. Illness**	
<2 yrs	302 (13%)
2–5 yrs	413 (17%)
5–10 yrs	453 (19%)
10 yrs+	994 (42%)
(Missing)	201 (8.5%)
**Psych. Comorb.**	
No	1,813 (77%)
Yes	549 (23%)
(Missing)	1 (< 0.1%)
**Suicide**	
0	1,635 (69%)
1	337 (14%)
2	104 (4.4%)
3	66 (2.8%)
4+	75 (3.2%)
(Missing)	146 (6.2%)
**Addictions**	
Nil	979 (41%)
Behav.	46 (1.9%)
Subst.	1,171 (50%)
Both	71 (3.0%)
(Missing)	96 (4.1%)
**Phys. Comorb.**	
No	1,752 (74%)
Yes	580 (25%)
(Missing)	31 (1.3%)
**Phys. Rx**	
No	1,958 (83%)
Yes	366 (15%)
(Missing)	39 (1.7%)
**Forensic Hx**	
No	1,910 (81%)
Yes	345 (15%)
(Missing)	108 (4.6%)
**Referrer**	
Pub. HC	1,743 (74%)
Pr. HC	299 (13%)
Soc. W.	54 (2.3%)
Pat.	102 (4.3%)
Other	85 (3.6%)
(Missing)	80 (3.4%)

^*1*^ n (%); Median (Q1, Q3)

Legend. dipl., diploma; HS, High-School; Bach., Bachelor’s degree; Mast., Master’s degree; rel., relationship; Div./Wid., Divorced/Widowed; Gp H., Group Home; Fam. H., Family Home; Pers. H., Personal Home; Hless, Homeless; Empl., Employed; reg., regular; spec., specialized; Unempl., Unemployed; Dis Work. Benef., Disability Worker Beneficiary; FGA, first generation antipsychotic; SGA, second generation antipsychotic; Dur. Illness, Duration of illness; <2 yrs, less than 2 years;10 yrs+, 10 years or more; Psych., Psychiatric; Comorb., Comorbidities; Phys., Physical; 4+, 4 or more; Behav., Behavioral; Subst., Substance; Rx, Treatment; Hx, History; Pub. HC, Public HealthCare; Pr. HC, Private HealthCare; Soc. W., Social Worker; Pat., Patient

### Performance metrics

**Fig. 1:**
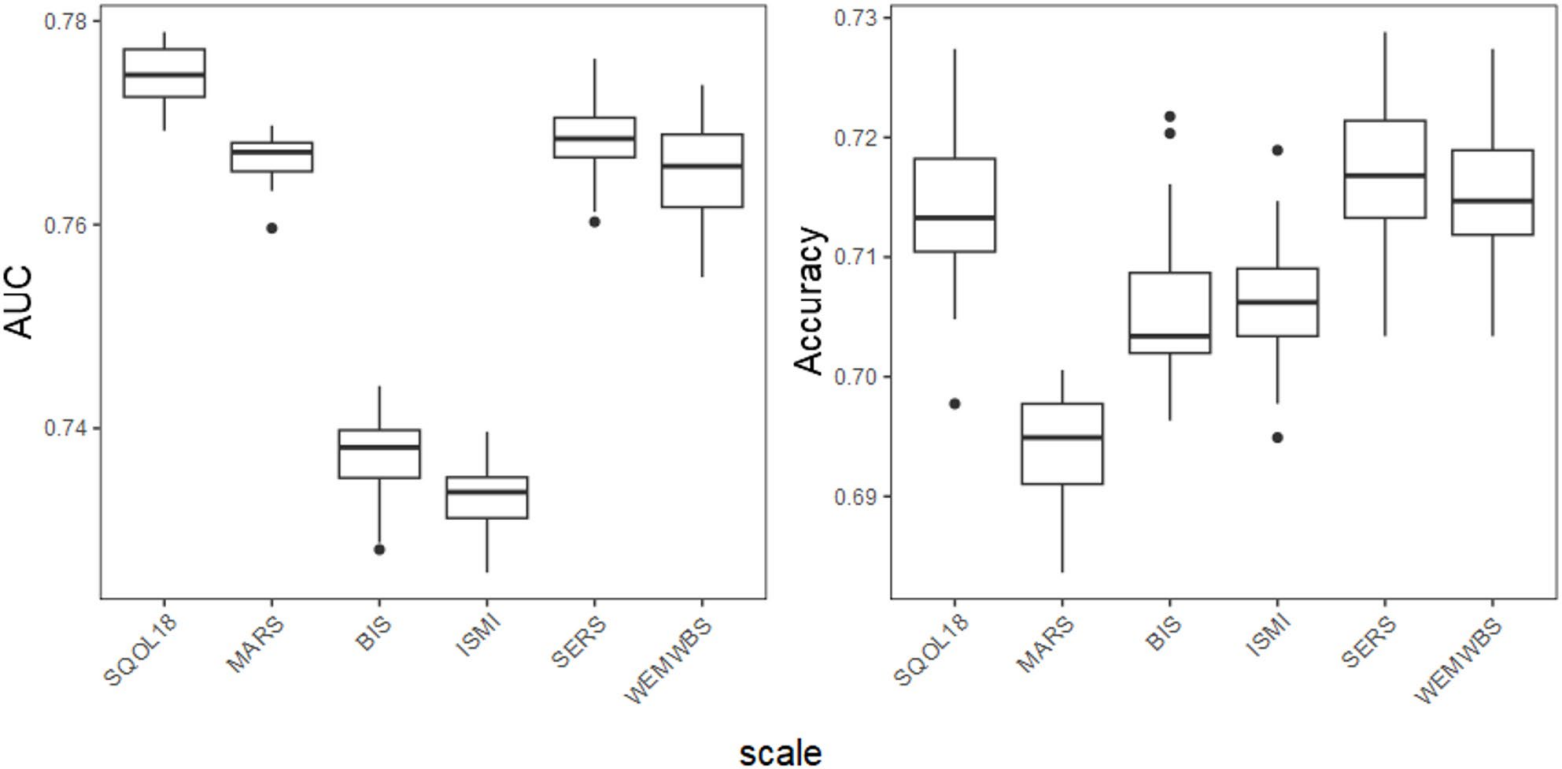
Performance metrics of the predictive models. For each patient-reported outcome, the area under the receiver operating characteristic curve (AUC) and accuracy are shown as boxplots reflecting variability across imputed datasets. Performance metrics were computed independently for each imputed dataset using a holdout test set, with results aggregated across all imputations. The box represents the interquartile range (IQR), spanning from the 25th percentile (Q1) to the 75th percentile (Q3); the horizontal line inside the box indicates the median (50th percentile); the whiskers extend to the smallest and largest values within 1.5 times the IQR from Q1 and Q3, respectively. Legend. SQOL18, Schizophrenia Quality of Life questionnaire (short form); MARS: Medication Adherence Rating Scale; BIS: Birchwood Insight Scale; ISMI: Internalized Stigma of Mental Illness; SERS, Self-Esteem Rating Scale; WEMBS: Warwick-Edinburgh Mental Well-being Scale

Figure 1 shows the performance metrics across 30 imputed datasets for each of the six PRO scales. Averaged AUC ranged from 0.73 (ISMI, SD: 0.003) to 0.78 (SQoL18, SD: 0.003). Accuracies ranged from 0.69 (MARS, SD: 0.004) to 0.72 (SERS, SD: 0.006). Supplementary Figs. 1 and 2 illustrate the cross-validated risks for the basis learners and the contribution of each basis learner to the SuperLearner ensemble across imputed datasets for each of the six PRO scales. Almost all basis learners played a significant role in the ensemble. As a point of comparison, the AUC values from our SuperLearner ensemble were at most 0.02 higher than those from the regularized regression basis learner (without automatic feature preselection).

Using a 10-fold cross-validation procedure rather than a single train/test split, and applying a random forest algorithm to impute missing values within a single dataset, the averaged AUC ranged from 0.74 (BIS, SD: 0.05) to 0.77 (MARS, SD: 0.05), while accuracies ranged from 0.69 (MARS, SD: 0.03) to 0.72 (WEMWBS, SD: 0.04).

### Influence of predictors

**Fig. 2:**
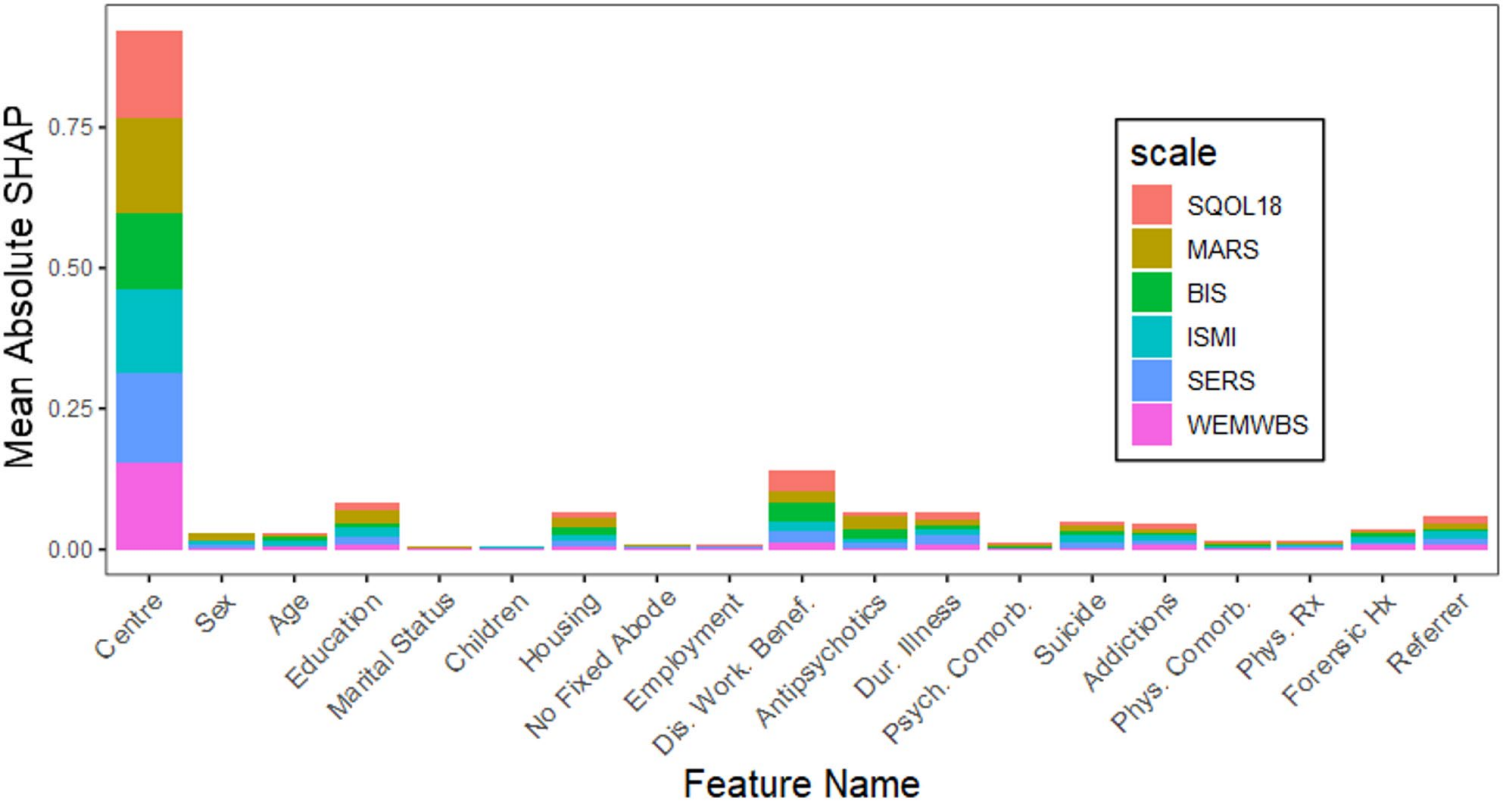
Mean absolute SHAP value for each feature and each rehab outcome measure. Mean absolute SHAP (SHapley Additive exPlanation) values are represented for each feature, stacked over each rehabilitation scale. These values indicate the strength of each feature’s importance in the model’s predictions. Legend. SQOL18, Schizophrenia Quality of Life questionnaire (short form); MARS: Medication Adherence Rating Scale; BIS: Birchwood Insight Scale; ISMI: Internalized Stigma of Mental Illness; SERS, Self-Esteem Rating Scale; WEMBS: Warwick-Edinburgh Mental Well-being Scale; Dis. Work. Benef., Disability Worker Beneficiary; Dur., Duration; Psych., Psychiatric; Comorb, Comorbidities; Phys, Physical; Rx, treatment; Hx, history

Figure 2 shows the mean absolute SHAP value for each feature and each rehab outcome measure (equivalent to an importance plot). Center of data collection was by far the most influential predictor. Other than Center, the ten most influential predictors were: being a disabled worker beneficiary; educational attainment; housing status; duration of illness; antipsychotic medication; origin of the referrer; number of suicide attempts; addictions comorbidity; having a forensic history; and sex. Note that a post hoc analysis using a model based solely on the study center produced AUCs ranging from 0.71 (ISMI) to 0.77 (WEMWBS) and accuracies ranging from 0.66 (MARS) to 0.74 (WEMWBS). The absolute differences in AUC between the full model and the center-only model ranged from − 0.003 (WEMWBS) to 0.03 (MARS), while differences in absolute accuracy ranged from − 0.03 (WEMWBS) to 0.03 (MARS).

**Fig. 3:**
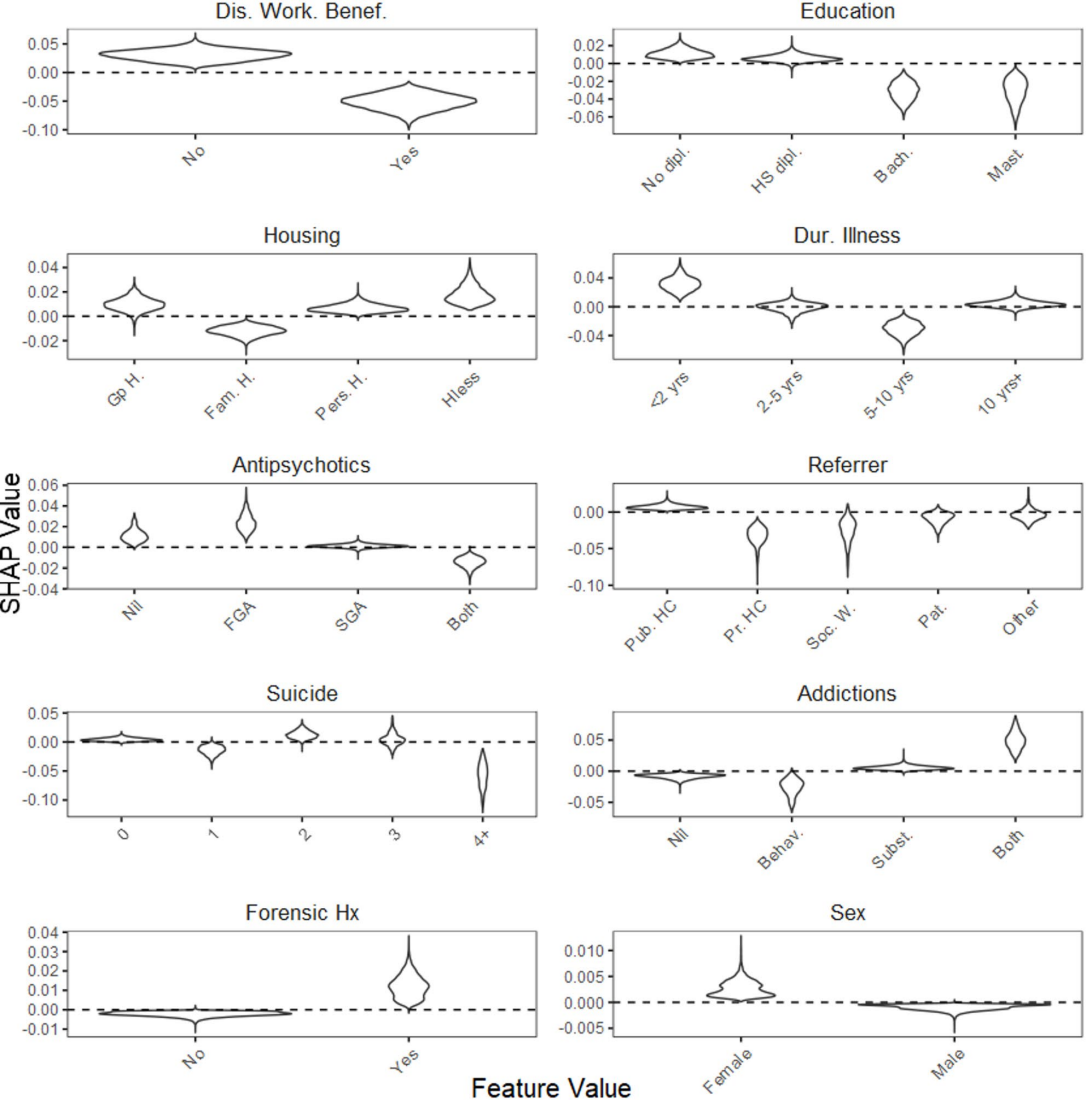
One-Way SHAP dependence plot of the 10 most influential features for the SQoL18. The distribution of SHAP values is shown for each feature value across imputed datasets. Higher SHAP values indicate a greater likelihood of missing data. Legend. SQoL18, Schizophrenia Quality of Life questionnaire (short form); Dis Work. Benef., Disability Worker Beneficiary; dipl., diploma; HS, High-School; Bach., Bachelor’s degree; Mast., Master’s degree; Gp H., Group Home; Fam. H., Family Home; Pers. H., Personal Home; Hless, Homeless; Dur., Duration; <2 yrs, less than 2 years; 10yrs+, 10 years or more; FGA, first generation antipsychotic; SGA, second generation antipsychotic; Pub. HC, Public HealthCare; Pr. HC, Private HealthCare; Soc. W., Social Worker; Pat., Patient; 4+, 4 or more; Behav., Behavioral; Subst., Substance; Hx, History

**Fig. 4:**
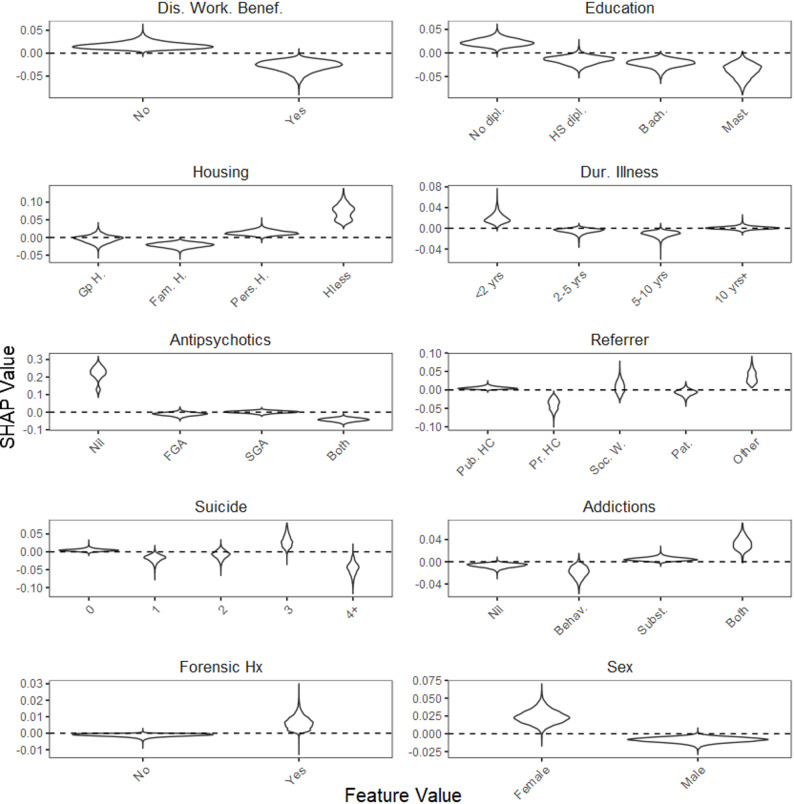
One-Way SHAP dependence plot of the 10 most influential features for the MARS. The distribution of SHAP values is shown for each feature value across imputed datasets. Higher SHAP values indicate a greater likelihood of missing data. Legend. MARS, Medication Adherence Rating Scale; Dis Work. Benef., Disability Worker Beneficiary; dipl., diploma; HS, High-School; Bach., Bachelor’s degree; Mast., Master’s degree; Gp H., Group Home; Fam. H., Family Home; Pers. H., Personal Home; Hless, Homeless; Dur., Duration; <2 yrs, less than 2 years; 10yrs+, 10 years or more; FGA, first generation antipsychotic; SGA, second generation antipsychotic; Pub. HC, Public HealthCare; Pr. HC, Private HealthCare; Soc. W., Social Worker; Pat., Patient; 4+, 4 or more; Behav., Behavioral; Subst., Substance; Hx, History

Figures 3 and 4 show the distribution of SHAP values for these 10 most important features (other than Center) for two PRO scales, the SQoL18 and the MARS, respectively. Similarities were observed between both scales. For instance, non-disability worker beneficiaries, individuals without a high school diploma, those experiencing homelessness, those with a short duration of illness, individuals with both substance and behavioral addictions, those with a forensic history, and female participants, were more likely to have missing data. Conversely, being referred by a private practitioner, having a history of four or more suicide attempts, and receiving treatment with both first-generation and second-generation antipsychotics were associated with a decreased likelihood of missing data. Slight differences were also observed. For instance, patients referred by social workers showed a decreased likelihood of missing values on the SQoL18, while patients not prescribed antipsychotics exhibited a higher likelihood of missing values on the MARS.

Other scales exhibited broadly similar SHAP value distributions, with subtle variations between individual measures (Supplementary Figs. 3–6). For instance, not being on antipsychotics increased the likelihood of missing BIS and ISMI data, while referrals by a social worker decreased the likelihood of missing SERS and WEMWBS data.

For reference, Supplementary Figs. 7–12 illustrate the distribution of SHAP values for less influential features across all six PRO scales. In addition, the coefficients of the main predictors (other than centers) derived from our regularized regression algorithm are presented in Supplementary Tables 2–7.

## Discussion

This study aimed to assess the predictability of missing PRO data in psychosocial rehabilitation, focusing on patients with schizophrenia. Our findings demonstrate that missing data can be predicted with good AUC and accuracy metrics. The most significant predictor of missing data was the center of data collection. Patient characteristics, while contributory, explained a considerably smaller proportion of missing data occurrence. Finally, the set of most impactful predictors exhibited only slight variations across different measures.

The predictability of missing data in rehabilitation outcome scales emerged as a significant finding in our study, which has two important implications. Firstly, the predictability of missing data (AUC > 0.5) strongly suggests that occurrences of missing data are not completely random, but rather systematic in nature. This finding aligns with a substantial body of literature in the field [1–4], while contradicting some studies that have posited a more random distribution of missing values [30, 31]. The systematic differences between individuals with and without missing data underscore the potential for non-response bias, a specific form of selection bias, in research studies, should the inherent differences between respondents and non-respondents not be taken into account.

Our findings are consistent with two possible missingness mechanisms. When data are Missing At Random (MAR), the probability that a PRO value is missing may depend on observed variables in the dataset, but not on the unobserved (missing) PRO value itself. In this scenario, missingness is systematic yet explained by observed data – it is conditionally random rather than absolutely random, meaning that missingness can be considered ignorable given the observed variables. However, the possibility of Missing Not At Random (MNAR) cannot be excluded. For instance, patients with lower quality of life may be less likely to complete related questionnaires. Because this mechanism depends on unobserved data, it cannot be directly tested and remains a potential source of bias in prediction and imputation.

Secondly, the predictive capability of our models offers a practical advantage in clinical settings. Drawing from our study findings, we have developed an internal predictive tool that integrates key components of our ensemble model and incorporates all identified features to estimate the likelihood of missing data for each new patient. Once the risk of missing data is assessed, clinicians and researchers can implement targeted interventions to improve retention, such as more intensive follow-up of high risk individuals. This in turn may contribute to having less missing patient-reported psychosocial rehabilitation outcome data. Improved completeness of PRO data enhances the accuracy and representativeness of treatment outcome evaluations, supporting more evidence-based clinical decisions. From a public health perspective, these gains can facilitate better monitoring of psychosocial rehabilitation services and inform the allocation of resources toward interventions that most effectively promote recovery and well-being.

Among the various predictors of missing data, the center of data collection emerged as the most influential factor. This finding underscores the significant role that institutional characteristics and practices play in data completeness. The observed center-specific variations in missing data rates can be attributed to a complex interplay of factors. Some centers may struggle with staffing shortages or have an organizational culture that does not prioritize comprehensive documentation, leading to higher rates of missing data. Conversely, centers affiliated with teaching hospitals often benefit from dedicated resources for data collection and may have clinical leaders who emphasize the importance of complete datasets, resulting in lower rates of missing information.

Furthermore, the strategies employed by different centers to improve data collection can significantly impact missing data rates. For instance, some institutions have implemented electronic reminder systems to enhance PRO completion rates [32]. Other centers may adopt a more focused approach, where they prioritize a selected number of scales rather than attempting to collect data on an extensive set [33]. This strategy can lead to maximized data collection for certain scales while minimizing it for others.

A model based solely on the study center achieved predictive performance relatively close to that of the full model, suggesting that the added value of demographic or clinical predictors was marginal. That said, patients’ characteristics, while less influential than center characteristics, still played a notable role in the occurrence of missing values. These include being a disability worker beneficiary, education level, housing status, duration of illness, the type of antipsychotic treatments received, the origin of the referrer, history of suicide attempts, addiction comorbidity, forensic history, and sex. Upon examining these factors, discernible patterns emerged that may have potential explanations. Individuals with a recent onset of illness and non-disability worker beneficiaries may not have established care routines, making consistent data collection challenging. Those without a high school diploma may have reduced health literacy, making it more challenging to complete complex surveys. Female participants and individuals experiencing homelessness often face competing priorities, such as family obligations, or the need to find accommodation, which can interfere with study participation. Individuals with substance and behavioral addictions may be more likely to have missing data due to lower levels of motivation, unstable lifestyles, or cognitive impairments caused by substance use. People with a forensic history might be more hesitant to disclose personal information due to stigma or legal concerns. Conversely, individuals with a history of four or more suicide attempts and those receiving treatment with both first- and second-generation antipsychotics may have more intensive follow-up care, potentially involving more frequent clinical visits and data collection opportunities.

In predictive analytics, it is important to remember that establishing and interpreting relationships between predictors and outcomes can be challenging as these relationships are not specifically targeted in the original study design. Nonetheless, our findings offer valuable insights into features associated with missing values. Addressing these identified predictors could help tackle the root causes of missing data, paving the way for public health interventions to reduce service withdrawal and loss to follow-up. Ultimately, this approach could enhance both the quality of patient care and the robustness of research in the field.

Our analysis revealed that predictors of missing values can vary in their influence depending on the specific scale being considered. We examined two paradigmatic examples: the Schizophrenia Quality of Life scale (SQoL18), and the Medication Adherence Rating Scale (MARS). While both measures demonstrated relatively similar performance and overall predictor importance, slight differences emerged, likely due to the inherent characteristics of each scale. For instance, the MARS exhibited a higher likelihood of missing values among patients not prescribed antipsychotics, as the scale is not administered to individuals not undergoing treatment. Conversely, the SQoL18 showed decreased likelihood of missing values when patients were referred by social workers. This may be due to the fact that when social workers serve as referrers, they often prioritize quality of life and well-being – concerns inherently linked to social functioning – making these outcomes less likely to be overlooked.

### Strengths and limitations

Our study has a number of strengths. Primarily, our dataset boasts a substantial number of observations and a wide array of predictors, providing a robust foundation for analysis. Also, we employed sophisticated modeling techniques to achieve optimal prediction of missing data occurrence, utilizing an ensemble of various basis learners with a cross-validation strategy and an adaptive scheme to optimize parameters for each basis learner. Finally, we leveraged the state-of-the-art concept of Shapley values to investigate the influence of individual predictors in the genesis of missing values, offering nuanced insights into the factors contributing to data incompleteness.

Nevertheless, our study is not without limitations. First, despite achieving a relatively high AUC, the performance could have been enhanced with the inclusion of additional background data, such as motivational factors, personality traits, and racial/ethnic information. Second, our study lacks insight into the specific reasons for missingness, a gap that future iterations of our database may address. Third, we encountered some missing data in our predictor matrix, necessitating imputation. We excluded two potential predictors that showed high variability in their influence when imputed. Fourth, the generalizability of our results may be limited to schizophrenia populations in rehabilitation settings, potentially not extending to other mental health populations such as those with autism spectrum or bipolar affective disorders, or to schizophrenia populations outside of rehabilitation contexts. In addition, given the heterogeneity in missingness across centers, our model may not generalize well beyond our network of rehabilitation centers. Nonetheless, it appears promising for context-specific monitoring. Fifth, our ensemble approach achieved only a modest gain of 0 to 0.02 AUC compared to the simpler regularized regression model. This marginal improvement may be due to non-linearities and interactions between variables explaining only a small proportion of missingness. Coefficients obtained from the regularized regression were consistent with those identified through the SHAP values analysis but are more straightforward to interpret.

## Conclusion

Our study demonstrates that missing data in schizophrenia PRO databases can be predicted with good performance. Institutional factors emerged as the most influential predictors of missingness, while patient characteristics – including age, illness duration, addiction comorbidity, education level, and disability beneficiary status – showed smaller effects. Our developed machine learning algorithm may be used in clinical settings to identify at-risk individuals and in turn enhance data collection and retention in rehabilitation care.

## Supplementary Information

Below is the link to the electronic supplementary material.

Supplementary Material 1

## Data Availability

The data supporting the findings of this study cannot be made publicly available. Our analysis plan was published and amended on GitHub before the actual analysis was carried out (https://github.com/gbarbalat/predict-missing-REHABase). Our code for this analysis is also available in the same repository.
