# Supplementary material for: Anticipating incomplete patient-reported outcomes in schizophrenia: a machine learning approach to predict the occurrence of missing data
Source: Health Qual Life Outcomes. 2026 Mar 14;24:52. doi: 10.1186/s12955-026-02511-1 (PMC13104463; doi:10.1186/s12955-026-02511-1)
Supplement: Supplementary file 1 — Supplementary Material 1 [file 12955_2026_2511_MOESM1_ESM.docx]

Supplementary Materials

Anticipating incomplete patient-reported outcomes in schizophrenia: a machine learning approach to predict the occurrence of missing data

Table of Contents

[Supplementary Table 1. Characteristics of the population across the imputed datasets 2](#__RefHeading___Toc7066_1776164764)

[Supplementary Table 2. Coefficients of the 10 most influential features (other than centers) obtained with the regularized regression basis learner across imputed datasets for the SQoL18 4](#__RefHeading___Toc6703_4111488710)

[Supplementary Table 3. Coefficients of the 10 most influential features (other than centers) obtained with the regularized regression basis learner across imputed datasets for the MARS 6](#__RefHeading___Toc6705_4111488710)

[Supplementary Table 4. Coefficients of the 10 most influential features (other than centers) obtained with the regularized regression basis learner across imputed datasets for the BIS 8](#__RefHeading___Toc6707_4111488710)

[Supplementary Table 5. Coefficients of the 10 most influential features (other than centers) obtained with the regularized regression basis learner across imputed datasets for the ISMI 10](#__RefHeading___Toc6709_4111488710)

[Supplementary Table 6. Coefficients of the 10 most influential features (other than centers) obtained with the regularized regression basis learner across imputed datasets for the SERS 12](#__RefHeading___Toc6711_4111488710)

[Supplementary Table 7. Coefficients of the 10 most influential features (other than centers) obtained with the regularized regression basis learner across imputed datasets for the WEMWBS 14](#__RefHeading___Toc6713_4111488710)

[Supplementary Figure 1. Cross-validated risks for the basis learners 16](#__RefHeading___Toc7068_1776164764)

[Supplementary Figure 2. Contribution of each basis learner to the SuperLearner ensemble 17](#__RefHeading___Toc7070_1776164764)

[Supplementary Figure 3. One-Way SHAP dependence plot of the 10 most influential features for the BIS 18](#__RefHeading___Toc7072_1776164764)

[Supplementary Figure 4. One-Way SHAP dependence plot of the 10 most influential features for the ISMI 19](#__RefHeading___Toc7074_1776164764)

[Supplementary Figure 5. One-Way SHAP dependence plot of the 10 most influential features for the SERS 20](#__RefHeading___Toc7076_1776164764)

[Supplementary Figure 6. One-Way SHAP dependence plot of the 10 most influential features for the WEMWBS 21](#__RefHeading___Toc7078_1776164764)

[Supplementary Figure 7. One-Way SHAP dependence plot of the eight least influential features for the SQoL18 22](#__RefHeading___Toc7080_1776164764)

[Supplementary Figure 8. One-Way SHAP dependence plot of the eight least influential features for the MARS 23](#__RefHeading___Toc7082_1776164764)

[Supplementary Figure 9. One-Way SHAP dependence plot of the eight least influential features for the BIS 24](#__RefHeading___Toc7084_1776164764)

[Supplementary Figure 10. One-Way SHAP dependence plot of the eight least influential features for the ISMI 25](#__RefHeading___Toc7086_1776164764)

[Supplementary Figure 11. One-Way SHAP dependence plot of the eight least influential features for the SERS 26](#__RefHeading___Toc7088_1776164764)

[Supplementary Figure 12. One-Way SHAP dependence plot of the eight least influential features for the WEMWBS 27](#__RefHeading___Toc7090_1776164764)

# Supplementary Table 1. Characteristics of the population across the imputed datasets

| Variable | N min | N max | Pct min | Pct max |
| --- | --- | --- | --- | --- |
| Education |  |  |  |  |
| No dipl. | 1118 | 1128 | 47.3 | 47.7 |
| HS dipl. | 712 | 721 | 30.1 | 30.5 |
| Bach. | 375 | 386 | 15.9 | 16.3 |
| Mast. | 141 | 147 | 6 | 6.2 |
| Marital Status |  |  |  |  |
| In a rel. | 275 | 287 | 11.6 | 12.1 |
| Div./Wid. | 111 | 120 | 4.7 | 5.1 |
| Single | 1962 | 1974 | 83 | 83.5 |
| Children |  |  |  |  |
| No | 2084 | 2096 | 88.2 | 88.7 |
| Yes | 267 | 279 | 11.3 | 11.8 |
| Housing |  |  |  |  |
| Gp H. | 222 | 231 | 9.4 | 9.8 |
| Fam. H. | 956 | 968 | 40.5 | 41 |
| Pers. H. | 1075 | 1087 | 45.5 | 46 |
| Hless | 92 | 99 | 3.9 | 4.2 |
| No Fixed Abode |  |  |  |  |
| No | 2086 | 2097 | 88.3 | 88.7 |
| Past | 187 | 195 | 7.9 | 8.3 |
| Current | 75 | 86 | 3.2 | 3.6 |
| Employment |  |  |  |  |
| Empl.(reg.) | 134 | 145 | 5.7 | 6.1 |
| Empl.(spec.) | 49 | 53 | 2.1 | 2.2 |
| Unempl. | 2165 | 2178 | 91.6 | 92.2 |
| Dis. Work. Benef. |  |  |  |  |
| No | 1437 | 1482 | 60.8 | 62.7 |
| Yes | 881 | 926 | 37.3 | 39.2 |
| Antipsychotics |  |  |  |  |
| Nil | 106 | 111 | 4.5 | 4.7 |
| FGA | 181 | 185 | 7.7 | 7.8 |
| SGA | 1516 | 1525 | 64.2 | 64.5 |
| Both | 548 | 555 | 23.2 | 23.5 |
| Dur. Illness |  |  |  |  |
| <2 yrs | 319 | 344 | 13.5 | 14.6 |
| 2-5 yrs | 437 | 463 | 18.5 | 19.6 |
| 5-10 yrs | 482 | 505 | 20.4 | 21.4 |
| 10 yrs+ | 1082 | 1101 | 45.8 | 46.6 |
| Psych. Comorb. |  |  |  |  |
| No | 1813 | 1814 | 76.7 | 76.8 |
| Yes | 549 | 550 | 23.2 | 23.3 |
| Suicide |  |  |  |  |
| 0 | 1723 | 1756 | 72.9 | 74.3 |
| 1 | 350 | 369 | 14.8 | 15.6 |
| 2 | 106 | 118 | 4.5 | 5 |
| 3 | 66 | 75 | 2.8 | 3.2 |
| 4+ | 75 | 82 | 3.2 | 3.5 |
| Addictions |  |  |  |  |
| Nil | 1013 | 1033 | 42.9 | 43.7 |
| Behav. | 47 | 52 | 2 | 2.2 |
| Subst. | 1206 | 1223 | 51 | 51.8 |
| Both | 71 | 81 | 3 | 3.4 |
| Phys. Comorb. |  |  |  |  |
| No | 1770 | 1779 | 74.9 | 75.3 |
| Yes | 584 | 593 | 24.7 | 25.1 |
| Phys. Rx |  |  |  |  |
| No | 1995 | 2007 | 84.4 | 84.9 |
| Yes | 356 | 368 | 15.1 | 15.6 |
| Forensic Hx |  |  |  |  |
| No | 1983 | 1992 | 83.9 | 84.3 |
| Yes | 371 | 380 | 15.7 | 16.1 |
| Referrer |  |  |  |  |
| Pub. HC | 1786 | 1798 | 75.6 | 76.1 |
| Pr. HC | 307 | 320 | 13 | 13.5 |
| Soc. W. | 59 | 67 | 2.5 | 2.8 |
| Pat. | 103 | 110 | 4.4 | 4.7 |
| Other | 85 | 93 | 3.6 | 3.9 |

Legend. Pct, percentage; dipl., diploma; HS, High-School; Bach., Bachelor’s degree; Mast., Master’s degree; rel., relationship; Div./Wid., Divorced/Widowed; Gp H., Group Home; Fam. H., Family Home; Pers. H., Personal Home; Hless, Homeless; Empl., Employed; reg., regular; spec., specialized; Unempl., Unemployed; Dis Work. Benef., Disability Worker Beneficiary; FGA, first generation antipsychotic; SGA, second generation antipsychotic; Dur. Illness, Duration of illness; <2 yrs, less than 2 years;10 yrs+, 10 years or more; Psych., Psychiatric; Comorb., Comorbidities; Phys., Physical; 4+, 4 or more; Behav., Behavioral; Subst., Substance; Rx, Treatment; Hx, History; Pub. HC, Public HealthCare; Pr. HC, Private HealthCare; Soc. W., Social Worker; Pat., Patient.

# Supplementary Table 2. Coefficients of the 10 most influential features (other than centers) obtained with the regularized regression basis learner across imputed datasets for the SQoL18

| Feature | Mean | Lowest | Highest |
| --- | --- | --- | --- |
| Dis. Work. Benef. (Ref=No) |  |  |  |
| Yes | -0.37 | -0.44 | -0.3 |
| Education (Ref=No dipl.) |  |  |  |
| HS dipl. | 0 | -0.07 | 0 |
| Bach. | -0.2 | -0.42 | -0.12 |
| Mast. | -0.15 | -0.4 | -0.04 |
| Housing (Ref=Gp H.) |  |  |  |
| Fam. H. | -0.11 | -0.15 | -0.06 |
| Pers. H. | 0 | 0 | 0.05 |
| Hless | 0.01 | 0 | 0.23 |
| Dur. Illness (Ref=10 yrs+) |  |  |  |
| <2 yrs | 0.08 | 0 | 0.17 |
| 2-5 yrs | -0.01 | -0.25 | 0 |
| 5-10 yrs | -0.16 | -0.27 | -0.05 |
| Antipsychotics (Ref=Both) |  |  |  |
| Nil | 0.01 | 0 | 0.36 |
| FGA | 0.07 | 0.02 | 0.33 |
| SGA | 0 | 0 | 0.14 |
| Referrer (Ref= Pub. HC) |  |  |  |
| Pr. HC | -0.2 | -0.49 | -0.12 |
| Soc. W. | -0.08 | -0.74 | 0 |
| Pat. | -0.02 | -0.35 | 0 |
| Other | 0 | 0 | 0.09 |
| Suicide (Ref=0) |  |  |  |
| 1 | -0.02 | -0.14 | 0 |
| 2 | 0.01 | 0 | 0.13 |
| 3 | 0.01 | 0 | 0.17 |
| 4+ | -0.26 | -0.69 | -0.1 |
| Addictions (Ref=Behav.) |  |  |  |
| Nil | -0.05 | -0.1 | 0 |
| Subst. | 0.01 | 0 | 0.2 |
| Both | 0.24 | 0 | 0.83 |
| Forensic Hx (Ref=No) |  |  |  |
| Yes | 0.03 | 0 | 0.12 |
| Sex (Ref=Female) |  |  |  |
| Male | -0.01 | -0.19 | 0 |

The average, lowest and highest coefficients across imputed datasets are displayed.

Legend. SQoL18, Schizophrenia Quality of Life questionnaire (short form); Dis Work. Benef., Disability Worker Beneficiary; dipl., diploma; HS, High-School; Bach., Bachelor’s degree; Mast., Master’s degree; Gp H., Group Home; Fam. H., Family Home; Pers. H., Personal Home; Hless, Homeless; Dur., Duration; <2 yrs, less than 2 years; 10yrs+, 10 years or more; FGA, first generation antipsychotic; SGA, second generation antipsychotic; Pub. HC, Public HealthCare; Pr. HC, Private HealthCare; Soc. W., Social Worker; Pat., Patient; 4+, 4 or more; Behav., Behavioral; Subst., Substance; Hx, History.

# Supplementary Table 3. Coefficients of the 10 most influential features (other than centers) obtained with the regularized regression basis learner across imputed datasets for the MARS

| Feature | Mean | Lowest | Highest |
| --- | --- | --- | --- |
| Dis. Work. Benef. (Ref=No) |  |  |  |
| Yes | -0.24 | -0.31 | -0.17 |
| Education (Ref=No dipl.) |  |  |  |
| HS dipl. | -0.05 | -0.08 | 0 |
| Bach. | -0.05 | -0.1 | 0 |
| Mast. | -0.07 | -0.16 | 0 |
| Housing (Ref=Gp H.) |  |  |  |
| Fam. H. | -0.12 | -0.17 | -0.05 |
| Pers. H. | 0.03 | 0 | 0.1 |
| Hless | 0.15 | 0.04 | 0.24 |
| Dur. Illness (Ref=10 yrs+) |  |  |  |
| <2 yrs | 0.12 | 0.04 | 0.21 |
| 2-5 yrs | 0 | -0.03 | 0 |
| 5-10 yrs | -0.02 | -0.07 | 0 |
| Antipsychotics (Ref=Both) |  |  |  |
| Nil | 1.35 | 1.19 | 1.4 |
| FGA | 0 | 0 | 0 |
| SGA | 0.07 | 0.04 | 0.09 |
| Referrer (Ref= Pub. HC) |  |  |  |
| Pr. HC | -0.24 | -0.33 | -0.15 |
| Soc. W. | 0 | 0 | 0 |
| Pat. | 0 | 0 | 0 |
| Other | 0.03 | 0 | 0.14 |
| Suicide (Ref=0) |  |  |  |
| 1 | -0.01 | -0.1 | 0 |
| 2 | 0 | 0 | 0 |
| 3 | 0.06 | 0 | 0.26 |
| 4+ | -0.13 | -0.25 | 0 |
| Addictions (Ref=Behav.) |  |  |  |
| Nil | -0.04 | -0.09 | 0 |
| Subst. | 0 | 0 | 0 |
| Both | 0.11 | 0 | 0.27 |
| Forensic Hx (Ref=No) |  |  |  |
| Yes | 0.13 | 0.06 | 0.21 |
| Sex (Ref=Female) |  |  |  |
| Male | -0.09 | -0.11 | -0.07 |

The average, lowest and highest coefficients across imputed datasets are displayed.

Legend. MARS, Medication Adherence Rating Scale; Dis Work. Benef., Disability Worker Beneficiary; dipl., diploma; HS, High-School; Bach., Bachelor’s degree; Mast., Master’s degree; Gp H., Group Home; Fam. H., Family Home; Pers. H., Personal Home; Hless, Homeless; Dur., Duration; <2 yrs, less than 2 years; 10yrs+, 10 years or more; FGA, first generation antipsychotic; SGA, second generation antipsychotic; Pub. HC, Public HealthCare; Pr. HC, Private HealthCare; Soc. W., Social Worker; Pat., Patient; 4+, 4 or more; Behav., Behavioral; Subst., Substance; Hx, History.

# Supplementary Table 4. Coefficients of the 10 most influential features (other than centers) obtained with the regularized regression basis learner across imputed datasets for the BIS

| Feature | Mean | Lowest | Highest |
| --- | --- | --- | --- |
| Dis. Work. Benef. (Ref=No) |  |  |  |
| Yes | -0.38 | -0.43 | -0.32 |
| Education (Ref=No dipl.) |  |  |  |
| HS dipl. | 0 | 0 | 0 |
| Bach. | -0.05 | -0.1 | -0.01 |
| Mast. | -0.16 | -0.24 | -0.08 |
| Housing (Ref=Gp H.) |  |  |  |
| Fam. H. | -0.05 | -0.11 | 0 |
| Pers. H. | 0.09 | 0.02 | 0.16 |
| Hless | 0 | 0 | 0 |
| Dur. Illness (Ref=10 yrs+) |  |  |  |
| <2 yrs | 0.02 | 0 | 0.13 |
| 2-5 yrs | -0.01 | -0.1 | 0 |
| 5-10 yrs | -0.1 | -0.17 | -0.01 |
| Antipsychotics (Ref=Both) |  |  |  |
| Nil | 0.82 | 0.71 | 0.91 |
| FGA | 0 | 0 | 0 |
| SGA | 0.01 | 0 | 0.03 |
| Referrer (Ref= Pub. HC) |  |  |  |
| Pr. HC | -0.05 | -0.13 | 0 |
| Soc. W. | 0.01 | 0 | 0.11 |
| Pat. | 0 | -0.02 | 0 |
| Other | 0 | 0 | 0 |
| Suicide (Ref=0) |  |  |  |
| 1 | -0.11 | -0.19 | 0 |
| 2 | 0 | 0 | 0 |
| 3 | 0 | 0 | 0.09 |
| 4+ | -0.21 | -0.3 | 0 |
| Addictions (Ref=Behav.) |  |  |  |
| Nil | 0 | 0 | 0 |
| Subst. | 0 | 0 | 0 |
| Both | 0.2 | 0.06 | 0.33 |
| Forensic Hx (Ref=No) |  |  |  |
| Yes | 0.17 | 0.11 | 0.25 |
| Sex (Ref=Female) |  |  |  |
| Male | 0 | 0 | 0 |

The average, lowest and highest coefficients across imputed datasets are displayed.

Legend. BIS, Birchwood Insight Scale; Dis Work. Benef., Disability Worker Beneficiary; dipl., diploma; HS, High-School; Bach., Bachelor’s degree; Mast., Master’s degree; Gp H., Group Home; Fam. H., Family Home; Pers. H., Personal Home; Hless, Homeless; Dur., Duration; <2 yrs, less than 2 years; 10yrs+, 10 years or more; FGA, first generation antipsychotic; SGA, second generation antipsychotic; Pub. HC, Public HealthCare; Pr. HC, Private HealthCare; Soc. W., Social Worker; Pat., Patient; 4+, 4 or more; Behav., Behavioral; Subst., Substance; Hx, History.

# Supplementary Table 5. Coefficients of the 10 most influential features (other than centers) obtained with the regularized regression basis learner across imputed datasets for the ISMI

| Feature | Mean | Lowest | Highest |
| --- | --- | --- | --- |
| Dis. Work. Benef. (Ref=No) |  |  |  |
| Yes | -0.16 | -0.27 | -0.09 |
| Education (Ref=No dipl.) |  |  |  |
| HS dipl. | 0 | -0.03 | 0 |
| Bach. | -0.24 | -0.43 | -0.13 |
| Mast. | -0.16 | -0.41 | -0.06 |
| Housing (Ref=Gp H.) |  |  |  |
| Fam. H. | 0 | -0.01 | 0.03 |
| Pers. H. | 0.15 | 0.09 | 0.32 |
| Hless | 0.1 | 0.01 | 0.45 |
| Dur. Illness (Ref=10 yrs+) |  |  |  |
| <2 yrs | 0.03 | 0 | 0.18 |
| 2-5 yrs | 0 | 0 | 0.07 |
| 5-10 yrs | -0.13 | -0.22 | -0.05 |
| Antipsychotics (Ref=Both) |  |  |  |
| Nil | 0.22 | 0.13 | 0.64 |
| FGA | 0.02 | 0 | 0.27 |
| SGA | 0.01 | 0 | 0.16 |
| Referrer (Ref= Pub. HC) |  |  |  |
| Pr. HC | -0.26 | -0.45 | -0.19 |
| Soc. W. | -0.01 | -0.24 | 0.07 |
| Pat. | 0 | -0.06 | 0 |
| Other | -0.01 | -0.15 | 0 |
| Suicide (Ref=0) |  |  |  |
| 1 | -0.22 | -0.41 | -0.1 |
| 2 | 0.03 | 0 | 0.2 |
| 3 | 0.01 | 0 | 0.17 |
| 4+ | -0.2 | -0.6 | 0 |
| Addictions (Ref=Behav.) |  |  |  |
| Nil | 0.01 | -0.05 | 0.24 |
| Subst. | 0.03 | 0 | 0.36 |
| Both | 0.44 | 0.23 | 1.04 |
| Forensic Hx (Ref=No) |  |  |  |
| Yes | 0.33 | 0.24 | 0.55 |
| Sex (Ref=Female) |  |  |  |
| Male | -0.03 | -0.2 | 0 |

The average, lowest and highest coefficients across imputed datasets are displayed.

Legend. ISMI, Internalized Stigma of Mental Illness; Dis Work. Benef., Disability Worker Beneficiary; dipl., diploma; HS, High-School; Bach., Bachelor’s degree; Mast., Master’s degree; Gp H., Group Home; Fam. H., Family Home; Pers. H., Personal Home; Hless, Homeless; Dur., Duration; <2 yrs, less than 2 years; 10yrs+, 10 years or more; FGA, first generation antipsychotic; SGA, second generation antipsychotic; Pub. HC, Public HealthCare; Pr. HC, Private HealthCare; Soc. W., Social Worker; Pat., Patient; 4+, 4 or more; Behav., Behavioral; Subst., Substance; Hx, History.

# Supplementary Table 6. Coefficients of the 10 most influential features (other than centers) obtained with the regularized regression basis learner across imputed datasets for the SERS

| Feature | Mean | Lowest | Highest |
| --- | --- | --- | --- |
| Dis. Work. Benef. (Ref=No) |  |  |  |
| Yes | -0.22 | -0.32 | -0.13 |
| Education (Ref=No dipl.) |  |  |  |
| HS dipl. | 0 | -0.03 | 0 |
| Bach. | -0.13 | -0.31 | -0.03 |
| Mast. | -0.05 | -0.37 | 0 |
| Housing (Ref=Gp H.) |  |  |  |
| Fam. H. | -0.04 | -0.09 | 0 |
| Pers. H. | 0.1 | 0.03 | 0.26 |
| Hless | 0.03 | 0 | 0.3 |
| Dur. Illness (Ref=10 yrs+) |  |  |  |
| <2 yrs | 0.26 | 0.11 | 0.51 |
| 2-5 yrs | 0.01 | 0 | 0.12 |
| 5-10 yrs | -0.12 | -0.27 | -0.05 |
| Antipsychotics (Ref=Both) |  |  |  |
| Nil | 0.05 | 0 | 0.38 |
| FGA | 0.17 | 0.1 | 0.42 |
| SGA | 0.01 | 0 | 0.05 |
| Referrer (Ref= Pub. HC) |  |  |  |
| Pr. HC | -0.18 | -0.37 | -0.08 |
| Soc. W. | -0.05 | -0.58 | 0 |
| Pat. | 0 | 0 | 0.02 |
| Other | 0.15 | 0 | 0.41 |
| Suicide (Ref=0) |  |  |  |
| 1 | -0.06 | -0.22 | 0 |
| 2 | -0.02 | -0.17 | 0 |
| 3 | 0.03 | 0 | 0.38 |
| 4+ | -0.1 | -0.45 | 0 |
| Addictions (Ref=Behav.) |  |  |  |
| Nil | 0 | 0 | 0.12 |
| Subst. | 0.01 | 0 | 0.16 |
| Both | 0.41 | 0.16 | 0.82 |
| Forensic Hx (Ref=No) |  |  |  |
| Yes | 0.19 | 0.11 | 0.47 |
| Sex (Ref=Female) |  |  |  |
| Male | -0.05 | -0.23 | -0.01 |

The average, lowest and highest coefficients across imputed datasets are displayed.

Legend. SERS, Self-Esteem Rating Scale; Dis Work. Benef., Disability Worker Beneficiary; dipl., diploma; HS, High-School; Bach., Bachelor’s degree; Mast., Master’s degree; Gp H., Group Home; Fam. H., Family Home; Pers. H., Personal Home; Hless, Homeless; Dur., Duration; <2 yrs, less than 2 years; 10yrs+, 10 years or more; FGA, first generation antipsychotic; SGA, second generation antipsychotic; Pub. HC, Public HealthCare; Pr. HC, Private HealthCare; Soc. W., Social Worker; Pat., Patient; 4+, 4 or more; Behav., Behavioral; Subst., Substance; Hx, History.

# Supplementary Table 7. Coefficients of the 10 most influential features (other than centers) obtained with the regularized regression basis learner across imputed datasets for the WEMWBS

| Feature | Mean | Lowest | Highest |
| --- | --- | --- | --- |
| Dis. Work. Benef. (Ref=No) |  |  |  |
| Yes | -0.22 | -0.29 | -0.16 |
| Education (Ref=No dipl.) |  |  |  |
| HS dipl. | 0 | 0 | 0 |
| Bach. | -0.08 | -0.13 | 0 |
| Mast. | -0.03 | -0.1 | 0 |
| Housing (Ref=Gp H.) |  |  |  |
| Fam. H. | -0.02 | -0.06 | 0 |
| Pers. H. | 0 | 0 | 0 |
| Hless | 0 | 0 | 0 |
| Dur. Illness (Ref=10 yrs+) |  |  |  |
| <2 yrs | 0.1 | 0 | 0.2 |
| 2-5 yrs | 0 | 0 | 0 |
| 5-10 yrs | -0.06 | -0.16 | 0 |
| Antipsychotics (Ref=Both) |  |  |  |
| Nil | 0 | 0 | 0 |
| FGA | 0 | 0 | 0 |
| SGA | 0 | 0 | 0.01 |
| Referrer (Ref= Pub. HC) |  |  |  |
| Pr. HC | -0.21 | -0.32 | -0.13 |
| Soc. W. | -0.1 | -0.33 | 0 |
| Pat. | 0 | 0 | 0 |
| Other | 0.01 | 0 | 0.15 |
| Suicide (Ref=0) |  |  |  |
| 1 | 0 | 0 | 0 |
| 2 | 0 | 0 | 0.01 |
| 3 | 0 | 0 | 0.09 |
| 4+ | -0.14 | -0.36 | 0 |
| Addictions (Ref=Behav.) |  |  |  |
| Nil | 0 | 0 | 0 |
| Subst. | 0 | 0 | 0.01 |
| Both | 0.43 | 0.19 | 0.64 |
| Forensic Hx (Ref=No) |  |  |  |
| Yes | 0.17 | 0.09 | 0.28 |
| Sex (Ref=Female) |  |  |  |
| Male | 0 | 0 | 0 |

The average, lowest and highest coefficients across imputed datasets are displayed.

Legend. WEMWBS, Warwick-Edinburgh Mental Well-being Scale; Dis Work. Benef., Disability Worker Beneficiary; dipl., diploma; HS, High-School; Bach., Bachelor’s degree; Mast., Master’s degree; Gp H., Group Home; Fam. H., Family Home; Pers. H., Personal Home; Hless, Homeless; Dur., Duration; <2 yrs, less than 2 years; 10yrs+, 10 years or more; FGA, first generation antipsychotic; SGA, second generation antipsychotic; Pub. HC, Public HealthCare; Pr. HC, Private HealthCare; Soc. W., Social Worker; Pat., Patient; 4+, 4 or more; Behav., Behavioral; Subst., Substance; Hx, History.

# Supplementary Figure 1. Cross-validated risks for the basis learners


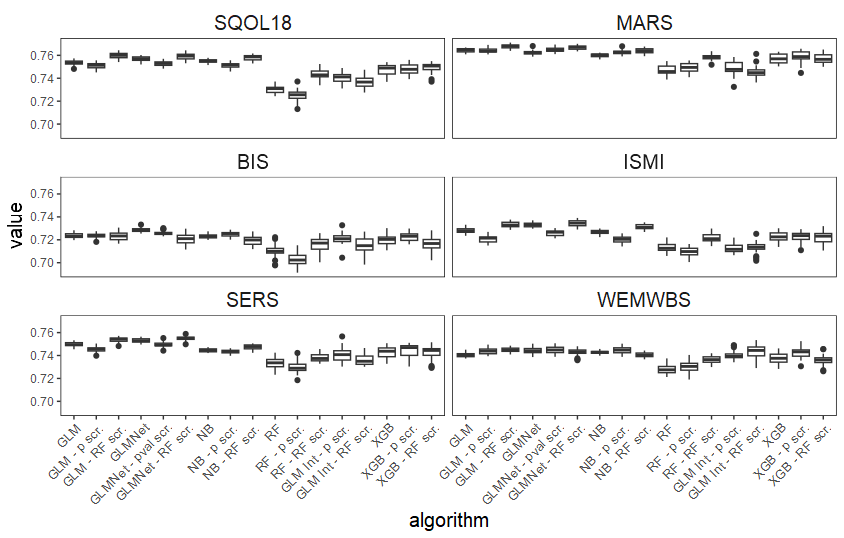


For each patient-reported outcome, cross-validated risks attributed to each basis learner are shown as boxplots reflecting variability across imputed datasets. The cross-validated risk values represent the area under the receiving operating curve (AUC). The box represents the interquartile range (IQR), spanning from the 25th percentile (Q1) to the 75th percentile (Q3); the horizontal line inside the box indicates the median (50th percentile); the whiskers extend to the smallest and largest values within 1.5 times the IQR from Q1 and Q3, respectively.

Legend. SQOL18, Schizophrenia Quality of Life questionnaire (short form); MARS: Medication Adherence Rating Scale; BIS: Birchwood Insight Scale; ISMI: Internalized Stigma of Mental Illness; SERS, Self-Esteem Rating Scale; WEMBS: Warwick-Edinburgh Mental Well-being Scale; GLM, Generalized Linear Model; GLM - p scr., Generalized Linear Model with p-value screening; GLM - RF scr., Generalized Linear Model with Random Forest screening; GLM Int - p scr., Generalized Linear Model with Interactions and p-value screening; GLM Int - RF scr., Generalized Linear Model with Interactions and Random Forest screening; GLMNet, Generalized Linear Model with regularization; GLMNet - pval scr., Generalized Linear Model with regularization and p-value screening; GLMNet - RF scr., Generalized Linear Model with regularization and Random Forest screening; NB, Naive Bayes; NB - p scr., Naive Bayes with p-value screening; NB - RF scr., Naive Bayes with Random Forest screening; RF, Random Forest; RF - p scr., Random Forest with p-value screening; RF - RF scr., Random Forest with Random Forest screening; XGB, eXtreme Gradient Boosting; XGB - p scr., eXtreme Gradient Boosting with p-value screening; XGB - RF scr., eXtreme Gradient Boosting with Random Forest screening

# Supplementary Figure 2. Contribution of each basis learner to the SuperLearner ensemble


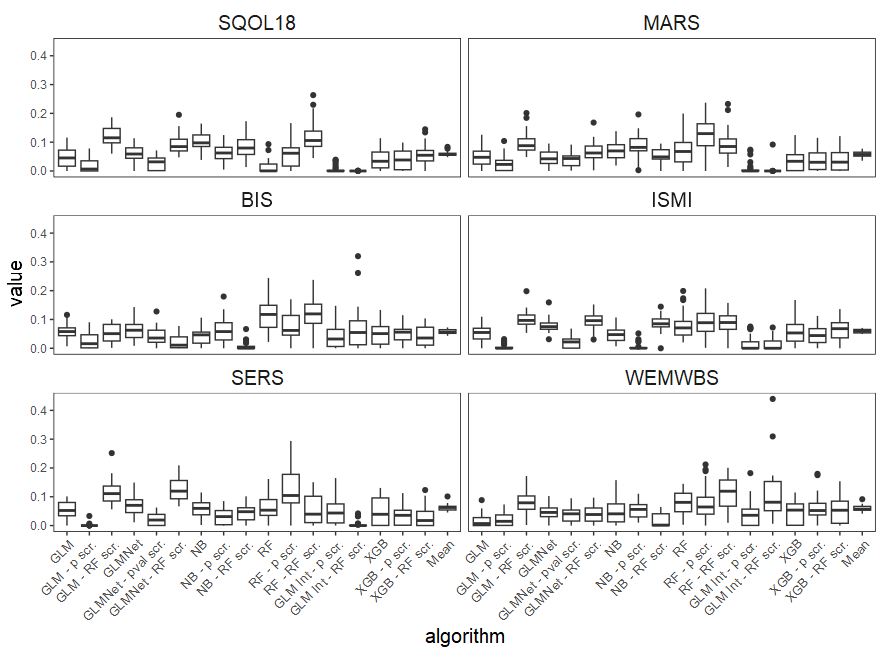


For each patient-reported outcome, coefficients attributed to each basis learner are shown as boxplots reflecting variability across imputed datasets. The box represents the interquartile range (IQR), spanning from the 25th percentile (Q1) to the 75th percentile (Q3); the horizontal line inside the box indicates the median (50th percentile); the whiskers extend to the smallest and largest values within 1.5 times the IQR from Q1 and Q3, respectively.

NB: The “mean” basis learner predicts outcomes using the average value of the target variable across all observations and is used as a benchmark.

Legend. SQOL18, Schizophrenia Quality of Life questionnaire (short form); MARS: Medication Adherence Rating Scale; BIS: Birchwood Insight Scale; ISMI: Internalized Stigma of Mental Illness; SERS, Self-Esteem Rating Scale; WEMBS: Warwick-Edinburgh Mental Well-being Scale; GLM, Generalized Linear Model; GLM - p scr., Generalized Linear Model with p-value screening; GLM - RF scr., Generalized Linear Model with Random Forest screening; GLM Int - p scr., Generalized Linear Model with Interactions and p-value screening; GLM Int - RF scr., Generalized Linear Model with Interactions and Random Forest screening; GLMNet, Generalized Linear Model with regularization; GLMNet - pval scr., Generalized Linear Model with regularization and p-value screening; GLMNet - RF scr., Generalized Linear Model with regularization and Random Forest screening; NB, Naive Bayes; NB - p scr., Naive Bayes with p-value screening; NB - RF scr., Naive Bayes with Random Forest screening; RF, Random Forest; RF - p scr., Random Forest with p-value screening; RF - RF scr., Random Forest with Random Forest screening; XGB, eXtreme Gradient Boosting; XGB - p scr., eXtreme Gradient Boosting with p-value screening; XGB - RF scr., eXtreme Gradient Boosting with Random Forest screening.

# Supplementary Figure 3. One-Way SHAP dependence plot of the 10 most influential features for the BIS


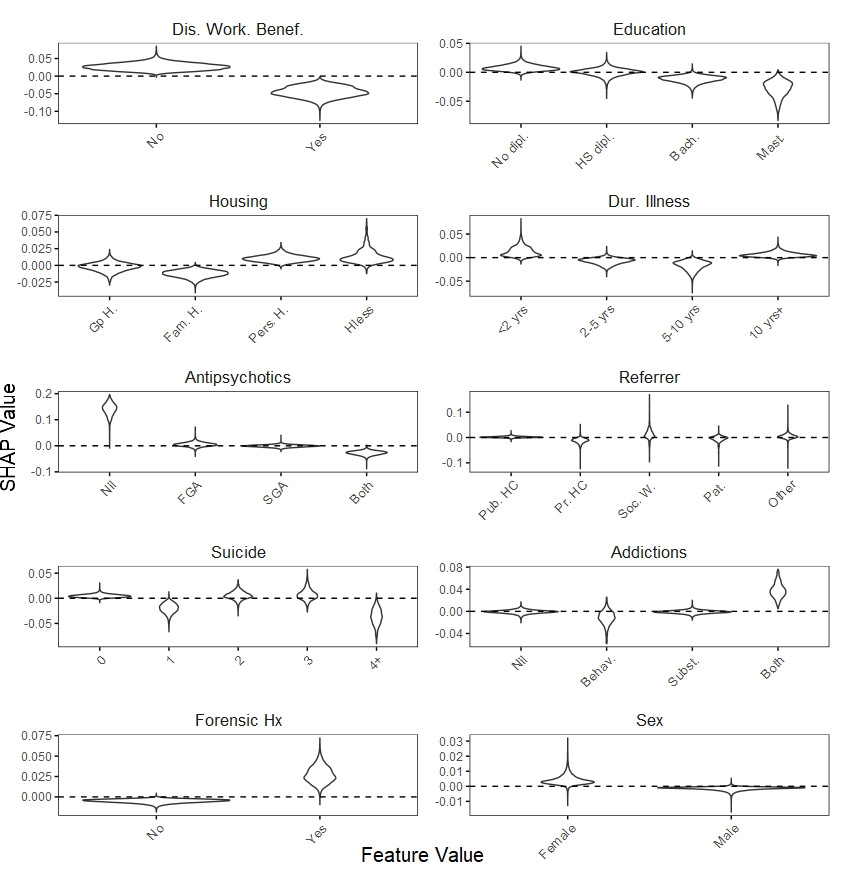


The distribution of SHAP values is shown for each feature value across imputed datasets. Higher SHAP values indicate a greater likelihood of missing data.

Legend. BIS, Birchwood Insight Scale; Dis Work. Benef., Disability Worker Beneficiary; dipl., diploma; HS, High-School; Bach., Bachelor’s degree; Mast., Master’s degree; Gp H., Group Home; Fam. H., Family Home; Pers. H., Personal Home; Hless, Homeless; Dur., Duration; <2 yrs, less than 2 years; 10yrs+, 10 years or more; FGA, first generation antipsychotic; SGA, second generation antipsychotic; Pub. HC, Public HealthCare; Pr. HC, Private HealthCare; Soc. W., Social Worker; Pat., Patient; 4+, 4 or more; Behav., Behavioral; Subst., Substance; Hx, History.

# Supplementary Figure 4. One-Way SHAP dependence plot of the 10 most influential features for the ISMI


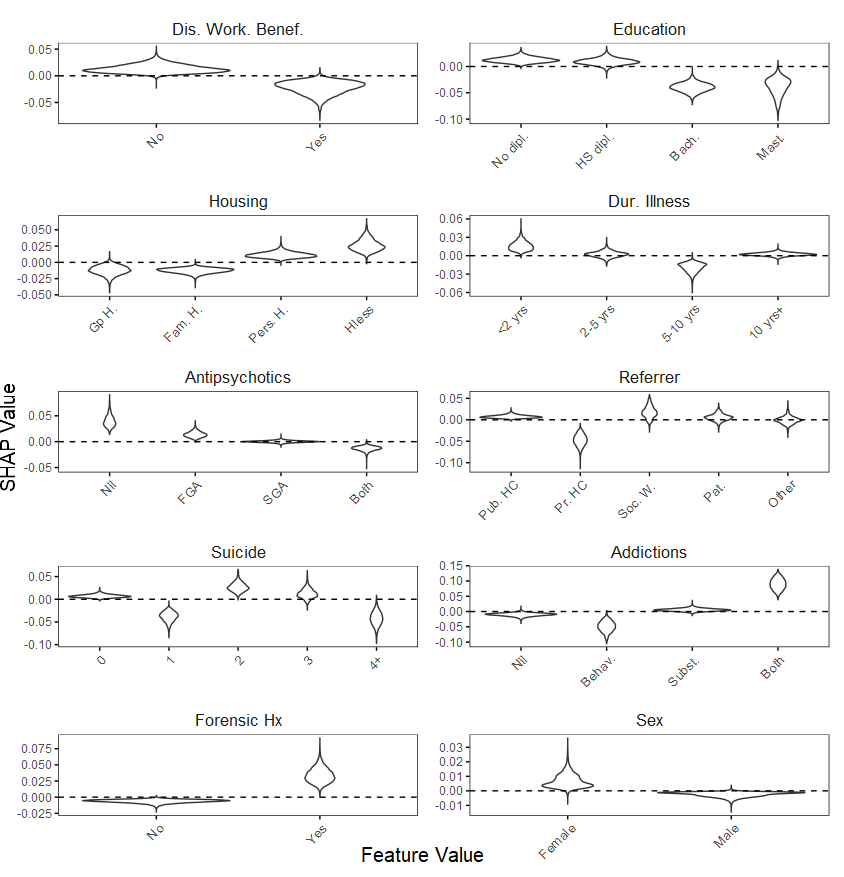


The distribution of SHAP values is shown for each feature value across imputed datasets. Higher SHAP values indicate a greater likelihood of missing data.

Legend. ISMI, Internalized Stigma of Mental Illness; Dis Work. Benef., Disability Worker Beneficiary; dipl., diploma; HS, High-School; Bach., Bachelor’s degree; Mast., Master’s degree; Gp H., Group Home; Fam. H., Family Home; Pers. H., Personal Home; Hless, Homeless; Dur., Duration; <2 yrs, less than 2 years; 10yrs+, 10 years or more; FGA, first generation antipsychotic; SGA, second generation antipsychotic; Pub. HC, Public HealthCare; Pr. HC, Private HealthCare; Soc. W., Social Worker; Pat., Patient; 4+, 4 or more; Behav., Behavioral; Subst., Substance; Hx, History.

# Supplementary Figure 5. One-Way SHAP dependence plot of the 10 most influential features for the SERS


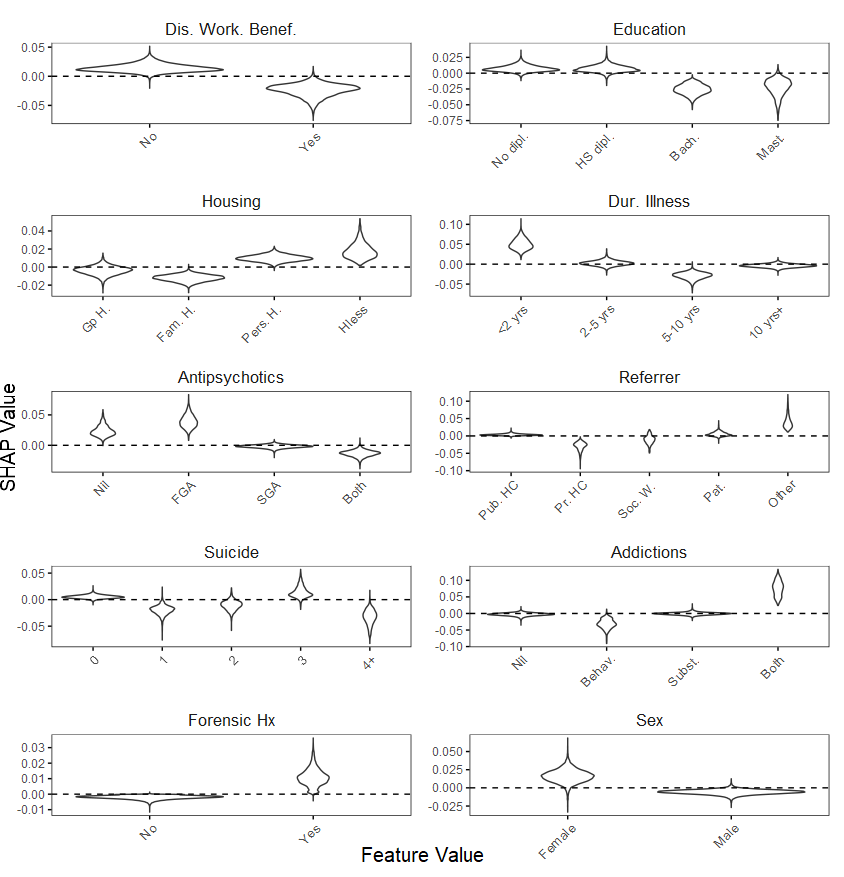


The distribution of SHAP values is shown for each feature value across imputed datasets. Higher SHAP values indicate a greater likelihood of missing data.

Legend. SERS, Self-Esteem Rating Scale; Dis Work. Benef., Disability Worker Beneficiary; dipl., diploma; HS, High-School; Bach., Bachelor’s degree; Mast., Master’s degree; Gp H., Group Home; Fam. H., Family Home; Pers. H., Personal Home; Hless, Homeless; Dur., Duration; <2 yrs, less than 2 years; 10yrs+, 10 years or more; FGA, first generation antipsychotic; SGA, second generation antipsychotic; Pub. HC, Public HealthCare; Pr. HC, Private HealthCare; Soc. W., Social Worker; Pat., Patient; 4+, 4 or more; Behav., Behavioral; Subst., Substance; Hx, History.

# Supplementary Figure 6. One-Way SHAP dependence plot of the 10 most influential features for the WEMWBS


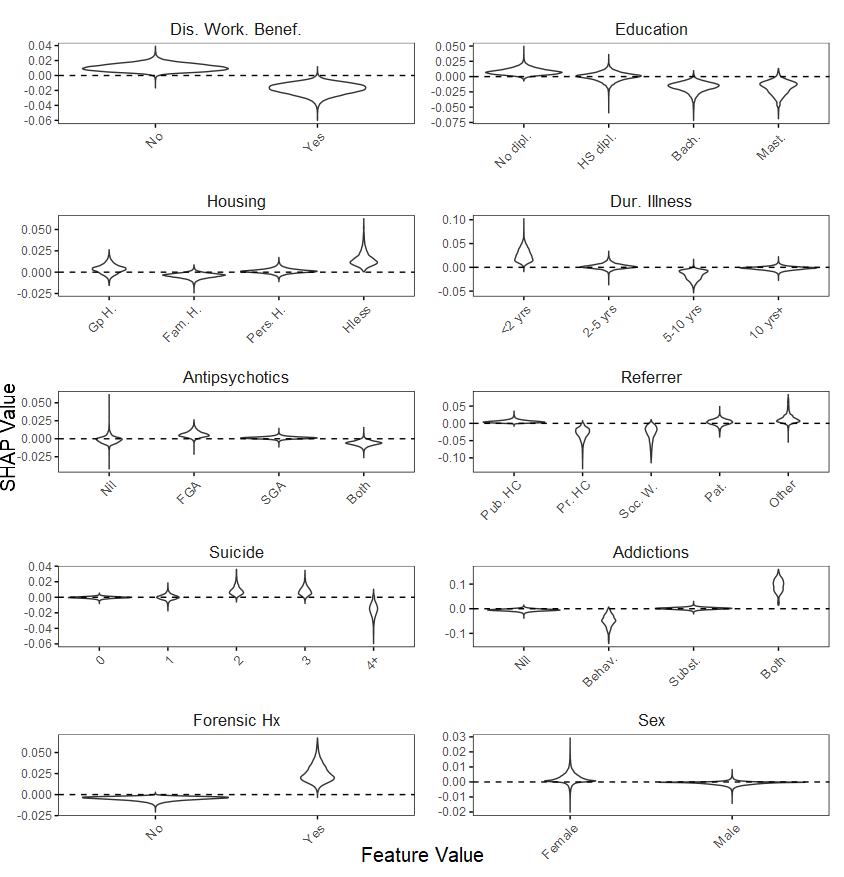


The distribution of SHAP values is shown for each feature value across imputed datasets. Higher SHAP values indicate a greater likelihood of missing data.

Legend. WEMWBS, Warwick-Edinburgh Mental Well-being Scale; Dis Work. Benef., Disability Worker Beneficiary; dipl., diploma; HS, High-School; Bach., Bachelor’s degree; Mast., Master’s degree; Gp H., Group Home; Fam. H., Family Home; Pers. H., Personal Home; Hless, Homeless; Dur., Duration; <2 yrs, less than 2 years; 10yrs+, 10 years or more; FGA, first generation antipsychotic; SGA, second generation antipsychotic; Pub. HC, Public HealthCare; Pr. HC, Private HealthCare; Soc. W., Social Worker; Pat., Patient; 4+, 4 or more; Behav., Behavioral; Subst., Substance; Hx, History.

# Supplementary Figure 7. One-Way SHAP dependence plot of the eight least influential features for the SQoL18


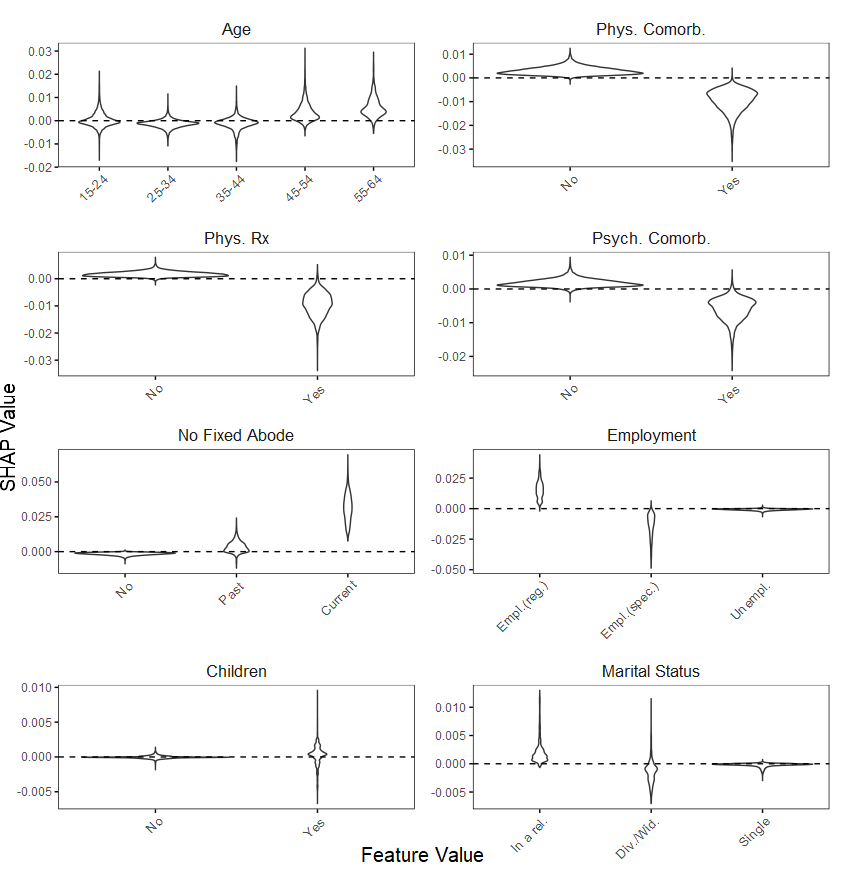


The distribution of SHAP values is shown for each feature value across imputed datasets. Higher SHAP values indicate a greater likelihood of missing data.

Legend. SQoL18, Schizophrenia Quality of Life questionnaire (short form); Phys., Physical; Comorb. Comorbidities; Rx, Treatment; Psych., Psychiatric; Empl., Employed; reg., regular; spec., specialized; Unempl., Unemployed; rel., relationship; Div./Wid., Divorced/Widowed.

# Supplementary Figure 8. One-Way SHAP dependence plot of the eight least influential features for the MARS


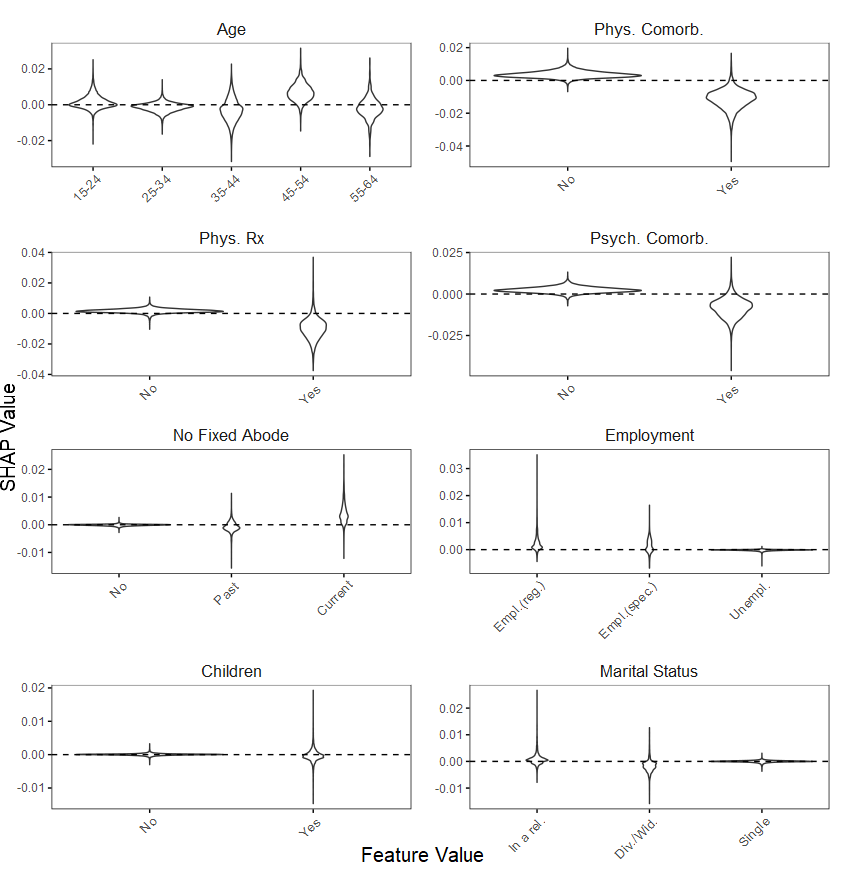


The distribution of SHAP values is shown for each feature value across imputed datasets. Higher SHAP values indicate a greater likelihood of missing data.

Legend. MARS, Medication Adherence Rating Scale; Phys., Physical; Comorb. Comorbidities; Rx, Treatment; Psych., Psychiatric; Empl., Employed; reg., regular; spec., specialized; Unempl., Unemployed; rel., relationship; Div./Wid., Divorced/Widowed.

# Supplementary Figure 9. One-Way SHAP dependence plot of the eight least influential features for the BIS


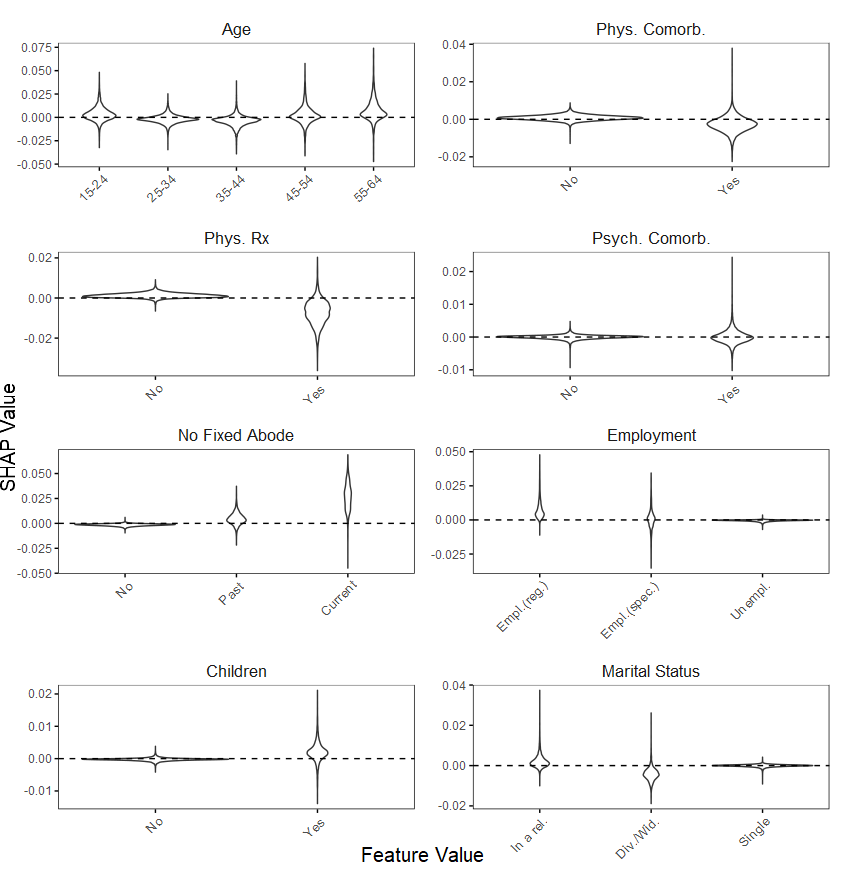


The distribution of SHAP values is shown for each feature value across imputed datasets. Higher SHAP values indicate a greater likelihood of missing data.

Legend. BIS, Birchwood Insight Scale; Phys., Physical; Comorb. Comorbidities; Rx, Treatment; Psych., Psychiatric; Empl., Employed; reg., regular; spec., specialized; Unempl., Unemployed; rel., relationship; Div./Wid., Divorced/Widowed.

# Supplementary Figure 10. One-Way SHAP dependence plot of the eight least influential features for the ISMI


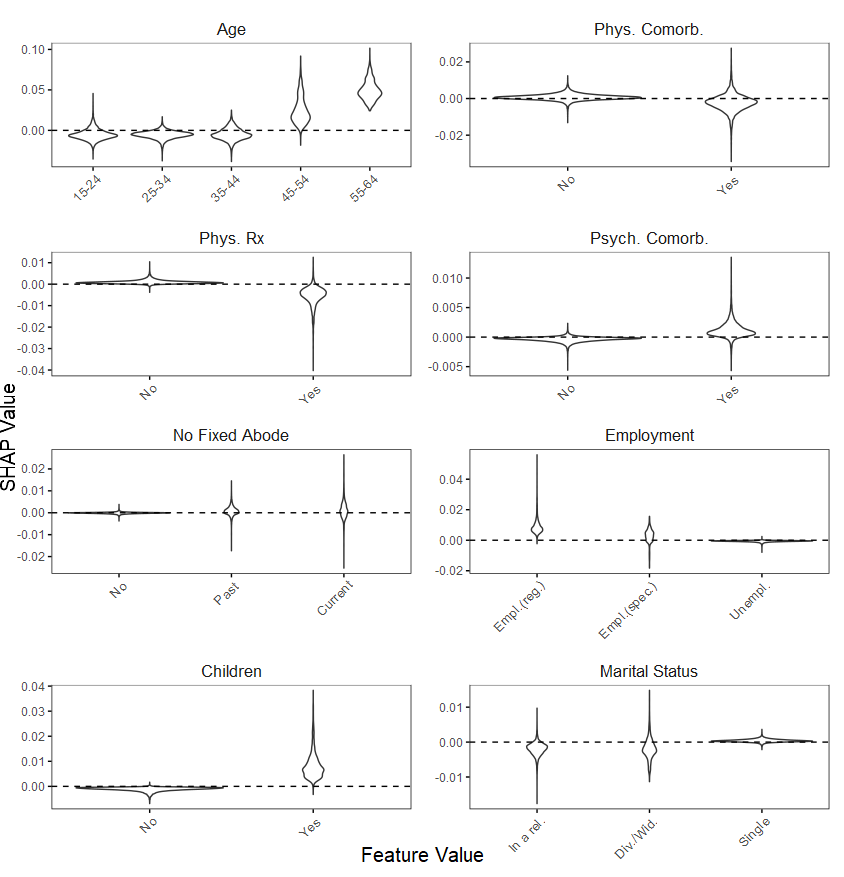


The distribution of SHAP values is shown for each feature value across imputed datasets. Higher SHAP values indicate a greater likelihood of missing data.

Legend. ISMI, Internalized Stigma of Mental Illness; Phys., Physical; Comorb. Comorbidities; Rx, Treatment; Psych., Psychiatric; Empl., Employed; reg., regular; spec., specialized; Unempl., Unemployed; rel., relationship; Div./Wid., Divorced/Widowed.

# Supplementary Figure 11. One-Way SHAP dependence plot of the eight least influential features for the SERS


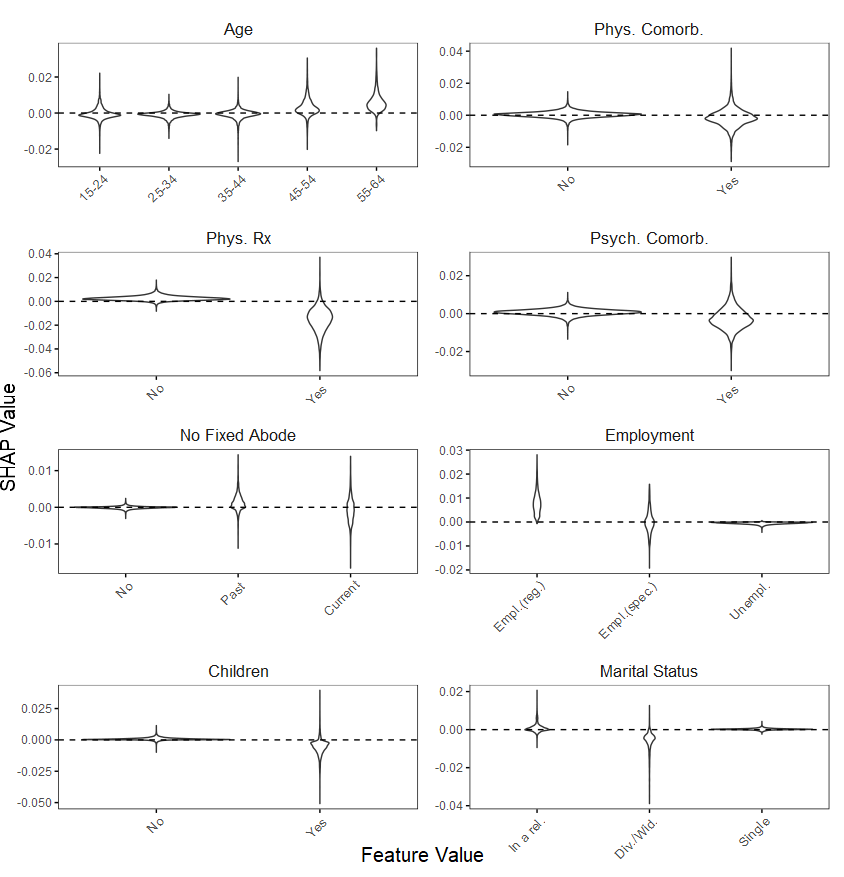


The distribution of SHAP values is shown for each feature value across imputed datasets. Higher SHAP values indicate a greater likelihood of missing data.

Legend. SERS, Self-Esteem Rating Scale; Phys., Physical; Comorb. Comorbidities; Rx, Treatment; Psych., Psychiatric; Empl., Employed; reg., regular; spec., specialized; Unempl., Unemployed; rel., relationship; Div./Wid., Divorced/Widowed.

# Supplementary Figure 12. One-Way SHAP dependence plot of the eight least influential features for the WEMWBS


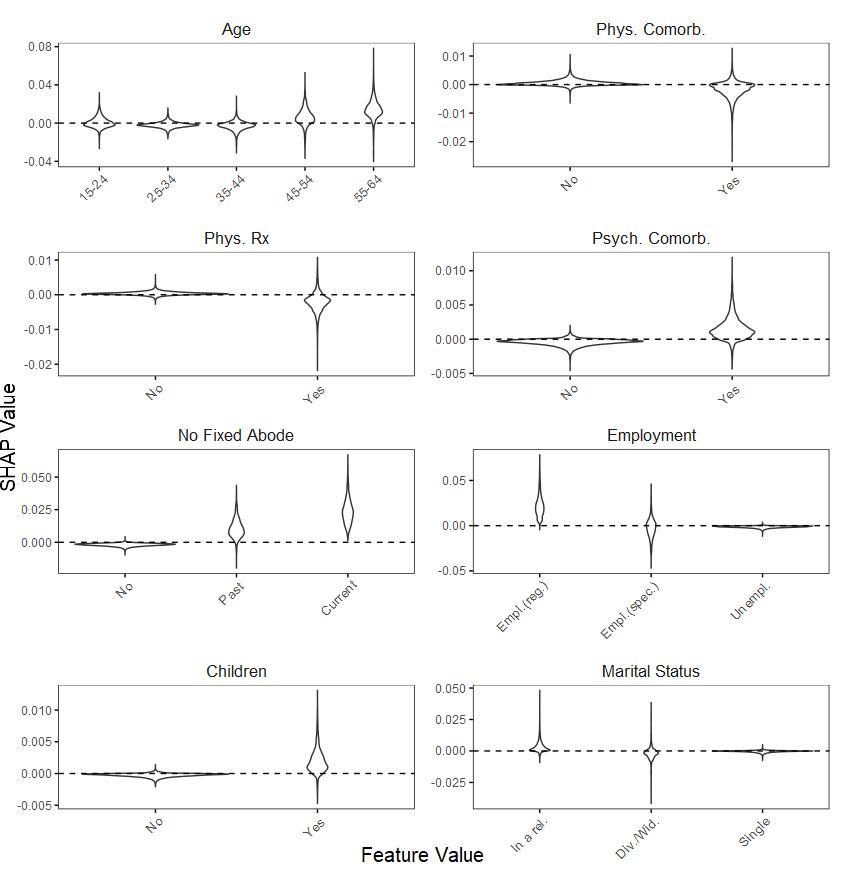


The distribution of SHAP values is shown for each feature value across imputed datasets. Higher SHAP values indicate a greater likelihood of missing data.

Legend. WEMWBS, Warwick-Edinburgh Mental Well-being Scale; Phys., Physical; Comorb. Comorbidities; Rx, Treatment; Psych., Psychiatric; Empl., Employed; reg., regular; spec., specialized; Unempl., Unemployed; rel., relationship; Div./Wid., Divorced/Widowed.
